# A comparative study of NETO1 and NETO2 on channel-opening kinetics of GluK2 kainate receptors

**DOI:** 10.1016/j.jbc.2025.110888

**Published:** 2025-11-04

**Authors:** Noah Saunders, Li Niu

**Affiliations:** Department of Chemistry and Center for Neuroscience Research, University at Albany, State University of New York, Albany, New York, USA

**Keywords:** glutamate receptors, kainate receptors, laser-pulse photolysis, kinetic mechanism of channel opening, NETO1/2

## Abstract

Neuropilin and tolloid–like proteins 1 and 2 (NETO1 and NETO2) are auxiliary subunits of kainate receptors, and GluK2 is a principal, pore-forming kainate receptor subunit. Kainate receptors are a subtype of ionotropic glutamate receptors. When coassembled with kainate receptors, NETO proteins are known to enhance the macroscopic current amplitude and affect channel properties such as channel desensitization rate. However, whether NETO proteins affect the rate of kainate receptor channel opening, which occurs in the microsecond time domain, is not known. Using a laser-pulse photolysis technique combined with whole-cell recording, we investigated the kinetic mechanism of channel opening of GluK2 homomeric receptors coexpressed with NETO1 and separately with NETO2 in human embryonic kidney-293 cells. We found that, as compared with GluK2 homomeric channels alone, NETO1 slows the channel-opening and channel-closing rate by ∼2-fold, whereas NETO2 slows these rates by ∼7-fold and ∼3-fold, respectively. Given that NETO2 also slows the rate of channel desensitization and reduces EC_50_ value more significantly than NETO1, our results show that NETO2 seems to be the more impactful auxiliary subunit on GluK2 homomeric channels.

Excitatory synaptic transmission is primarily mediated by ionotropic glutamate receptors in the central nervous system. Specifically, the neurotransmitter glutamate, released from presynaptic terminals, can bind to and activate three glutamate receptor subtypes: α-amino-3-hydroxy-5-methyl-4-isoxazolepropionic acid (AMPA) receptors, *N*-methyl-D-aspartate (NMDA) receptors, and kainate receptors (1, 2). The main function of AMPA receptors is to mediate the majority of fast neurotransmission, whereas NMDA receptors are responsible for synaptic plasticity. Unlike either AMPA or NMDA receptors, kainate receptors are expressed both presynaptically and postsynaptically. Postsynaptic kainate receptors mediate excitatory neurotransmission (3, 4), whereas presynaptic kainate receptors are involved in modulating the release of both excitatory and inhibitory neurotransmitters (4, 5, 6, 7, 8, 9). Dysregulation and dysfunction of kainate receptors have been implicated in a variety of neurological disorders, such as pain, epilepsy, and Huntington’s disease; therefore, kainate receptors represent an important target for therapeutic development (8, 10, 11, 12).

Kainate receptors are tetrameric assemblies of different combinations of the GluK1–5 subunits. Each of the GluK1–3 subunits can form homomeric channels, whereas GluK4 and GluK5 subunits only form heteromeric receptors with at least one of GluK1–3 (13, 14, 15, 16, 17). GluK4- and GluK5-containing channels have a higher affinity for glutamate than GluK1–3 (17, 18). Neuronal kainate receptors are known to contain high-affinity kainate receptor subunits (19) and also interact with auxiliary subunits, termed as NETO1 and NETO2, originally copurified with GluK2/3 from native tissue (20, 21, 22, 23). NETOs are single-pass CUB (complement C1r/C1s, Uegf, Bmp1) domain–containing transmembrane proteins (24). NETO1 and NETO2 are known to modulate the pore-forming kainate receptor subunits by affecting receptor trafficking, surface expression, and gating properties (24, 25). For example, NETO2 slows deactivation and desensitization but accelerates recovery from desensitization and attenuates polyamine block of calcium-permeable kainate receptors (20, 21, 23, 26, 27). However, whether NETO1 and NETO2 affect the rate of channel opening of kainate receptors is not known. The rate of channel opening defines the speed by which a kainate receptor opens its ion-channel pore, in response to binding of glutamate, by transitioning from the closed-channel to the open-channel state. The rate constant for channel opening, together with the rate of channel closing, is considered one of the most fundamental properties that defines a channel (28, 29).

The challenge to measure the rate of channel opening in response to glutamate binding to kainate receptors is that a kinetic technique must provide a sufficient time resolution since the channel opens in the submillisecond time domain but desensitizes on the millisecond scale. Commonly used solution exchange techniques do not offer a suitable time resolution to separate channel desensitization from channel-opening reaction. In this study, we used a laser-pulse photolysis technique to release glutamate from its biologically inert “caged glutamate” precursor (*i.e.*, 4-methoxy-7-nitroindolinyl glutamate) with a time constant of ∼30 μs (28, 29) and characterized the rate constants of the glutamate-induced channel opening for GluK2/NETO1 and GluK2/NETO2 receptors expressed in human embryonic kidney (HEK)-293 cells. The results from this study have provided an understanding of the functional impact of both NETO1 and NETO2 on the rate of channel opening of GluK2 receptors. Using GluK2 as a single receptor would allow us to tell the difference, if any, in differential impact between the two NETO proteins on the channel-opening kinetics of GluK2. Knowing the rate constant will enable a more quantitative prediction of the time course of the open channel as a function of neurotransmitter concentration, which determines the change in transmembrane voltage and in turn controls synaptic neurotransmission. In addition, characterization of the rate constants for the kinetic mechanism of channel opening will also provide clues for mechanism-based design of therapeutic compounds to treat neurological diseases involving native kainate receptors coassembled with different NETO proteins.

## Results

### Coexpression of kainate receptors with NETOs in HEK-293 cells

**Figure 1:**
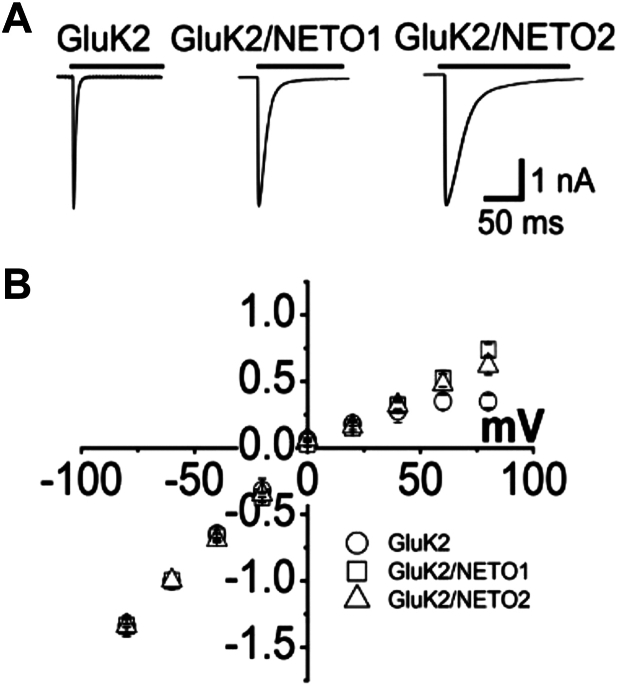
**Representative whole-cell current response and reduction of GluK2 inward rectification of the current–voltage relationship by NETO1/2.***A*, representative whole-cell current responses to glutamate from HEK-293 cells expressing GluK2, GluK2/NETO1, and GluK2/NETO2. Here, 10 mM glutamate was applied for 300 ms (*upper bar*) to cell voltage clamped at −60 mV. *B*, current–voltage relationships for GluK2, GluK2/NETO1, and GluK2/NETO2. Peak current amplitudes were collected at 10 mM glutamate from −80 mV to +80 mV at 20 mV intervals and normalized to the response at −60 mV for each cell. Each data point was from an average of at least three cells. The standard deviation from mean is shown as the error bar. GluK2, kainate receptor subunit 2; HEK-293, human embryonic kidney 293 cell line; NETO1/2, neuropilin tolloid–like proteins 1 and 2.

The experiments we designed in this study started with transient expression of each of the three kainate receptor channels, namely, GluK2, GluK2/NETO1, and GluK2/NETO2, in HEK-293 cells. When we coexpressed GluK2 with a NETO protein, we wanted to ensure that at least the majority of receptors expressed were coassembled with NETO proteins. To fulfill this requirement, we varied the ratio of the amount of the DNA plasmid encoding a NETO protein to the amount of the plasmid for GluK2 and then tested whole-cell response. As shown (Fig. 1*A*), coexpression of a NETO protein with GluK2 in HEK-293 cells produced different whole-cell current responses with different rates of channel desensitization. Specifically, we used the current–voltage (I–V) relationship to assess the GluK2/NETO1 expression. Expression of either NETO1 or NETO2 using a 1:2 plasmid ratio by weight resulted in attenuation of the inwardly rectified I–V relationship observed in GluK2 homomeric channels (Fig. 1*B*) (30). Further increases in the amount of a NETO plasmid did not alter the I–V relationship any further. Fisher and Mott (30) found that a series of three adjacent positively charged residues located near the intracellular side of the transmembrane domain in NETO1/2 are responsible for the decrease in inward rectification of the unedited form of GluK2. We also monitored the rate of channel desensitization for GluK2 in the absence and presence of a NETO protein. Again, further increase in the amount of the DNA plasmid ratio did not cause any further change of the rate constant of channel desensitization measured at any glutamate concentration we measured. Based on these measurements, we concluded that a weight ratio of 1:2 for GluK2-encoding to NETO-encoding DNA plasmids was sufficient to generate HEK-293 cells that predominantly expressed GluK2/NETO1 or GluK2/NETO2 channels (Fig. S1). This ratio was also consistent with the ratio published previously (30). Therefore, we used this plasmid ratio for the rest of the experiments in this study. It should be further noted that we checked the whole-cell current response at both −60 mV and +60 mV for every single cell we used to make sure that the cell did show the linear I–V relationship.

### NETO2 slowed GluK2 desensitization more than NETO1

**Figure 2:**
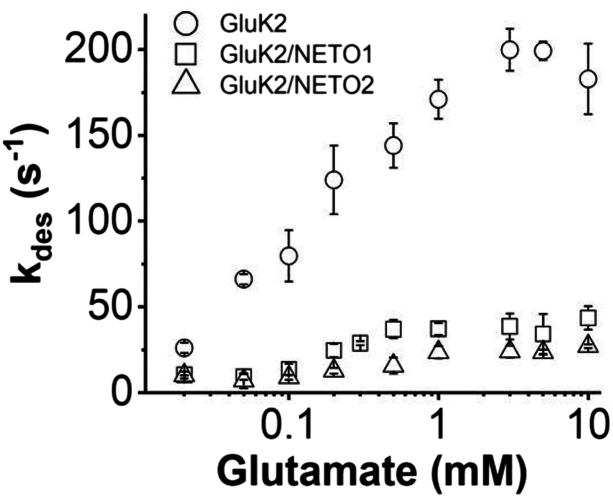
**Dependence of desensitization rate constant (***k*_*des*_**) on glutamate concentration (mM).** A first-order rate constant (*k*_des_) was adequate to describe >95% of the desensitization rate process for all three channel types: GluK2, GluK2/NETO1, and GluK2/NETO2. Each data point was from an average of at least three cells. Error bars represent standard deviation from mean. Maximal *k*_des_ values were analyzed using an unpaired *t* test. For each, n = 3 and *p* < 0.01 (see details in Table S8).

Following the confirmation of complex channel formation, we measured the effect of each NETO protein on the rate of channel desensitization, represented by the falling phase of a whole-cell current response to glutamate (Fig. 1). Specifically, we determined the rate constant of channel desensitization (*k*_des_) at various glutamate concentrations. By comparison of *k*_des_ among the three channel types (Fig. 2), several features were evident. First, the desensitization rate process of the whole-cell current followed a first-order rate for >98% of the process. This was true for the entire range of glutamate concentrations with and without NETOs. Second, the desensitization rate was significantly slower as observed in HEK-293 cells with coexpression of either NETO1 or NETO2, as compared with GluK2 alone (Figs. 1 and 2), suggesting a NETO protein had a significant impact on channel desensitization. Third, the rate constant of channel desensitization was glutamate concentration dependent in that the higher the glutamate concentration, the faster the rate of channel desensitization. The rate of channel desensitization reached the maximum for all three channel types at 3 mM glutamate. We found that coexpression of NETO1 with GluK2 and NETO2 with GluK2 exhibited the maximal *k*_des_ to 37 ± 6 s^−1^ and 27 ± 5 s^−1^, respectively. In contrast, GluK2 homomeric channels showed the maximum *k*_des_ of 199 ± 12 s^−1^. Therefore, coexpression of NETO1 and NETO2 with GluK2 resulted in a ∼5-fold and ∼7-fold decrease in the maximum *k*_des_, respectively. The maximum *k*_des_ value for GluK2/NETO1 and GluK2/NETO2 observed in this study (Fig. 2) was similar to the respective literature values (20, 31, 32, 33). The *k*_des_ for GluK2 homomeric channels without NETO was similar to the value we previously reported (29, 34). Overall, our results confirmed that the channel desensitization rate of either GluK2/NETO1 or GluK2/NETO2 was significantly slowed, as compared with the rate of homomeric GluK2 (20, 31, 32, 33). Our results further showed that NETO2 had a greater impact on slowing the desensitization of GluK2 than NETO1 (Table 1; all statistical analyses of these values in Table 1 are provided in supporting information Tables S7*A* and S8).

**Table 1:** Summary of the maximum desensitization rate constant, EC_50_, and *K*_1_ values for GluK2, **GluK2/NETO1**, and **GluK2/NETO2**

Receptor^a^	*k*_des_ (s^−1^)	EC_50_ (μM)	*K*_1_ (μM)
GluK2^b^	199 ± 12	255 ± 30	300 ± 210
GluK2/NETO1	37 ± 6∗	182 ± 17∗	210 ± 70
GluK2/NETO2	27 ± 5∗	58 ± 4∗	73 ± 8

a All values are listed as mean with SE.

b All statistical analyses are with GluK2 values, where ∗*p* < 0.05. The detailed analysis is provided in Tables S7, *A* and *B* for *K*_1_ and EC_50_; Table S8 is for *k*_des_ in supporting information.

### Impact of NETO1 and NETO2 on the GluK2 dose–response relationship

**Figure 3:**
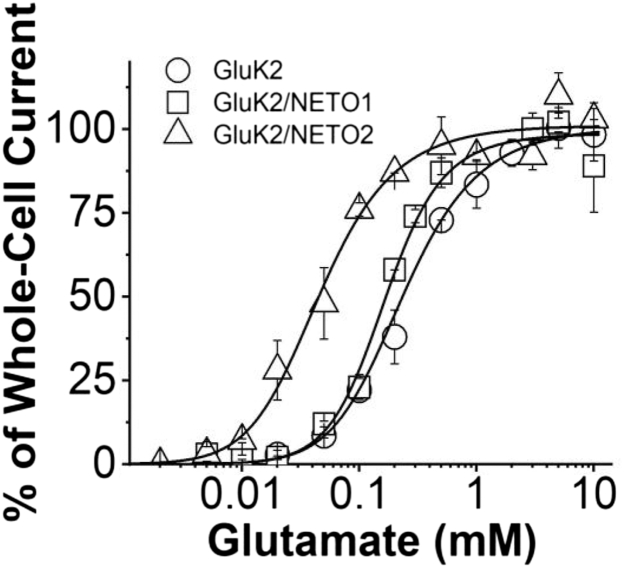
**Dose–response relationships of GluK2,GluK2/NETO1, andGluK2/NETO2.** Whole-cell current amplitudes in response to glutamate at concentrations ranging from 0.002 mM to 10 mM glutamate were measured relative to the amplitude at 0.2 mM glutamate. The maximal current amplitude was the average of the current response to 3, 5, and 10 mM glutamate and was set to be 100%. Nonlinear regression was used to fit the dose–response data using Equation 1 (see the Experimental procedures section). For the best fit judged by the reduced Chi-squared and adjusted *R*-squared values, and for each receptor when n = 2, *K*_1_ = 0.3 ± 0.21 mM, *Ф* = 0.48 ± 0.73, and *I*_*M*_*R*_*M*_ = 161 ± 84 for GluK2; *K*_1_ = 0.20 ± 0.05 mM, *Ф* = 0.32 ± 0.04, and *I*_*M*_*R*_*M*_ = 128 ± 12 for GluK2/NETO1; and *K*_1_ = 0.073 ± 0.01 mM, *Ф* = 0.20 ± 0.01, and *I*_*M*_*R*_*M*_ = 120 ± 5 for GluK2/NETO2. Additional data using nonlinear regression are provided as supporting information. The dose–response data were further analyzed using the Hill equation. The EC_50_ values for GluK2, GluK2/NETO1, and GluK2/NETO2 were estimated to be 255 ± 30 μM, 182 ± 17 μM, and 58 ± 4 μM, with the Hill coefficient being 1.7, 1.7, and 1.5, respectively. Each data point was from an average of at least three cells. Error bars represent standard deviation from mean. Statistical significance was determined with the Welch *t* test. For GluK2, GluK2/NETO1, and GluK2/NETO2, n = 27, n = 33, and n = 36, respectively (see details in Tables S7, *A* and *B*). For the EC_50_ values between GluK2 and GluK2 with a NETO, *p* < 0.05.

Next, we characterized the dose–response relationship for both GluK2/NETO1 and GluK2/NETO2. Coexpression of either NETO1 or NETO2 with GluK2 led to a left shift in their respective dose–response curves, as compared with the GluK2 control (Fig. 3). Using Equation 1 (see the Experimental procedures section), we analyzed the dose–response relationship by nonlinear regression to estimate the dissociation equilibrium constant (*K*_1_) and the number of ligand molecules that bind to and open the channel (*n*). By this method, the minimal number of glutamate molecules that could bind to and open the channel was found to be two (*i.e.*, *n* = 2) (a detailed nonlinear regression analysis is provided in Figs. S2–S4 and Tables S1*A*–S3*E*). The regression analysis was then refined using *n* = 2, and the results showed that *K*_1_ was 210 ± 70 μM for GluK2/NETO1 and 73 ± 8 μM for GluK2/NETO2, respectively, as compared with *K*_1_ of 300 ± 210 μM for GluK2 alone (all these values are summarized in Table 1).

We also analyzed the same dose–response data using the Hill equation and obtained that EC_50_ (or 50% of the maximum response) was 182 ± 107 μM for GluK2/NETO1 and 58 ± 4 μM for GluK2/NETO2, as compared with 255 ± 30 mM for GluK2 homomeric channels (Table 1 and Figs. S5–S7). These EC_50_ values were similar to those reported earlier (26, 31). The EC_50_ of GluK2 homomeric channels we determined in this study was also similar to the value we previously reported (29, 34). It should be noted that *K*_1_ and EC_50_ values are empirically similar. Our results confirmed that when NETO proteins were coassembled with GluK2, they promoted greater sensitivity of NETO-containing channels toward glutamate, as compared with GluK2 homomeric channels. A lower *K*_1_ or EC_50_ value suggested that a higher percentage of the channels in a receptor population could be opened at the same glutamate concentration, consistent with the notion that the channel was potentiated (20). Furthermore, the *K*_1_ and EC_50_ values of GluK2 channels decreased by ∼5- and ∼6-fold when GluK2 was coexpressed with NETO2, respectively; yet there was only a ∼1.4-fold decrease in those values when GluK2 was coexpressed with NETO1 (Table 1). This comparison showed that NETO2 had a greater impact on GluK2 in elevating glutamate sensitivity than NETO1.

### Impact of NETO1 and NETO2 on the channel-opening rate of GluK2 homomeric channels

**Figure 4:**
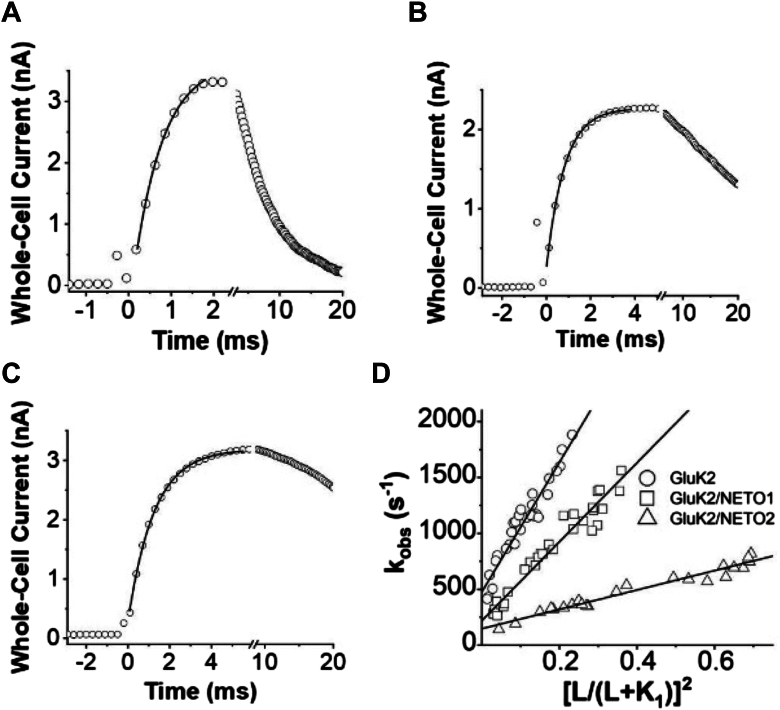
**The laser-pulse photolysis measurement for characterization of the channel-opening kinetics for GluK2,GluK2/NETO1, andGluK2/NETO2.***A*–*C*, representative whole-cell current trace generated by laser-pulse photolysis measurement of an HEK-293S cell expressing GluK2, GluK2/NETO1, and GluK2/NETO2. The laser was fired at time 0. In the three traces shown here, the photolytically released glutamate concentration was estimated to be 250 μM (see the Experimental procedures section). Note that the current is plotted opposite to the direction that is recorded. For clarity, we used one of every 40 data points for plotting. The *solid line* was a single exponential fit to the rising phase of the current response. The *k*_obs_ was calculated to be 1750 s^−1^, 1221 s^−1^, and 695 s^−1^ for GluK2, GluK2/NETO1, and GluK2/NETO2, respectively. *D*, determination of *k*_op_ and *k*_cl_ for each of the GluK2, GluK2/NETO1, and GluK2/NETO2 channels by linear regression assuming n = 2 using Equation 3. Each data point was a single *k*_obs_ value obtained at a particular concentration of glutamate. The resulting *k*_op_ and *k*_cl_ values were found to be (5.8 ± 0.3) × 10^3^ s^−1^ and (4.7 ± 0.3) × 10^2^ s^−1^ for GluK2, (3.5 ± 0.2) × 10^3^ s^−1^ and (2.2 ± 0.3) × 10^2^ s^−1^ for GluK2/NETO1, and (0.87 ± 0.04) × 10^3^ s^−1^ and (1.5 ± 0.2) × 10^2^ s^−1^ for GluK2/NETO2, respectively. Nonlinear regression analysis of *k*_obs_*versus* glutamate concentration using Equation 3 with various n values, and ethers, is provided in supporting information. Statistical significance in *k*_op_ and *k*_cl_ values between GluK2 and GluK2 with an NETO were analyzed using Welch *t* test (see details in Tables S7, *A* and *B*). For GluK2, GluK2/NETO1, and GluK2/NETO2, n = 28, n = 31, and n = 23, respectively. For all, *p* < 0.001.

**Figure 5:**
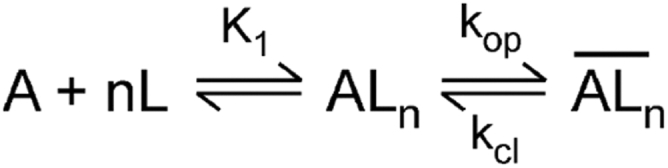
**A general, minimal mechanism of channel opening for kainate receptors.** Here, *A* represents the active, unliganded form of the receptor, *L* the ligand or glutamate, *AL*_*n*_ the closed-channel forms with *n* ligand molecules bound, and $\bar{{AL}_{n}}$ the open-channel state. The number of glutamate molecules that can bind to the receptor and open its channel, *n*, can be from 1 to 4, assuming that each subunit has one glutamate binding site and a maximum of four glutamate molecules could bind to a tetrameric receptor. It is further assumed that a ligand does not dissociate from the open channel state. The *k*_op_ and *k*_cl_ are the channel-opening and channel-closing rate constants, respectively. For simplicity and without contrary evidence, it is assumed that glutamate binds with equal affinity or *K*_1_, the intrinsic equilibrium dissociation constant, at all binding steps.

Using the laser-pulse photolysis technique combined with whole-cell recording (28, 29), we measured the rate of GluK2 channel opening with and without NETO proteins. This technique allowed us to investigate whether NETO proteins affected the channel-opening rate process of GluK2, and if so, whether NETO1 and NETO2 exerted the same functional impact. As shown (Fig. 4, *A–C*), the rise of whole-cell current response to glutamate that was photolytically liberated by the laser-pulse photolysis followed a first-order rate process (>95%) in all three channel types and in all the glutamate concentrations we measured. These results were consistent with the notion that the channel-opening step was rate limiting, or ligand binding was faster than channel opening. The observed first-order rate constant (*k*_obs_) was determined using Equation 2 (see the Experimental procedures section). The rate of the whole-cell current rise was slowed, or the rise time became longer, recorded from GluK2/NETO-expressing cells, as compared with HEK-293 cells that only expressed GluK2 (Fig. 4*D*). Furthermore, the observed rate of current rise was found to be faster at a higher concentration of photolytically released glutamate. These experimental observations can be explained using a minimal kinetic model (Fig. 5). Based on this model, the channel-opening (*k*_op_) and channel-closing (*k*_cl_) rate constants were determined from analysis of *k*_obs_ as a function of glutamate concentration using Equation 3 (see the Experimental procedures section). In our initial analysis of *k*_obs_ *versus* glutamate concentration, we ran nonlinear regression to evaluate all four parameters as in Equation 3 (the detailed procedure and the parameters used in this analysis and the output values generated are provided in Tables S3*A* and S4*C*). These analyses of each of the three channel types allowed us to conclude that the best fit produced *n* = 2 through nonlinear regression, suggesting that binding of two glutamate molecules per receptor was sufficient to open the GluK2 channel with or without NETOs. Based on *n* = 2, we then refined the fitting (Tables S4*A*–S6*C*) and produced *k*_op_ and *k*_cl_ values for all three types of channels using linear regression, as shown in Figure 4*D*. Specifically, *k*_op_ and *k*_cl_ were estimated to be (3.5 ± 0.2) × 10^3^ s^−1^ and (2.2 ± 0.3) × 10^2^ s^−1^ for GluK2/NETO1 and (0.87 ± 0.04) × 10^3^ s^−1^ and (1.5 ± 0.2) × 10^2^ s^−1^ for GluK2/NETO2, respectively. In contrast, *k*_op_ and *k*_cl_ were (5.8 ± 0.3) × 10^3^ s^−1^ and (4.7 ± 0.4) × 10^2^ s^−1^ for GluK2 homomeric channels, respectively (34, 35) (all these rate constants are summarized in Table 2; all statistical analyses of these values in Table 2 are provided in Supporting Information Tables S7, *A* and *B*).

**Table 2:** Summary of the channel-opening and channel-closing rate, constants, and channel-opening probability for GluK2, GluK2/NETO1, and GluK2/NETO2

Receptor^a^	*k*_op_ (×10^3^ s^−1^)	*k*_cl_ (×10^2^ s^−1^)	*P*_open_
GluK2^b^	5.8 ± 0.3	4.7 ± 0.4	0.96 ± 0.09
GluK2/NETO1	3.5 ± 0.2∗	2.2 ± 0.3∗	0.94 ± 0.15
GluK2/NETO2	0.87 ± 0.04∗	1.5 ± 0.2∗	0.86 ± 0.12

a All values are listed as mean with SE.

b All statistical analyses are with GluK2 values, where ∗*p* < 0.05. The detailed analysis is provided in Tables S7, *A* and *B* in supporting information.

The kinetic results showed that both NETO proteins slowed the channel-opening kinetics of GluK2. However, NETO2 slowed the rate of channel opening by >6-fold, whereas NETO1 only slowed the rate of channel opening for GluK2 channels by 1.7-fold. Both NETO proteins also slowed the rate of channel closing in that NETO2 reduced the rate of channel closing by threefold, whereas NETO1 caused just a twofold reduction of the rate of channel closing (Table 2). It should be noted that the *k*_cl_ for GluK2/NETO2 we determined agreed with the value previously reported from a single-channel measurement (20) (note that we converted the time constant, τ, which was reported to *k* for comparison, and is τ = 1/*k* for a first-order reaction). However, the *k*_cl_ for GluK2 homomeric channel we determined (*i.e.*, 470 s^−1^) is ∼2-fold larger than the channel-closing rate constant previously reported by the same study (20). Overall, our study revealed that each of the two NETO proteins, when coassembled with GluK2 subunit, formed the corresponding channels, which were slow to open and would stay open longer, once the channels were in the open conformation, as compared with GluK2 channels without NETOs. Furthermore, NETO2 had a significantly greater impact than NETO1 on GluK2 channel-opening rate process, especially in slowing the rate of channel opening.

We also determined the probability of channel opening (*P*_open_), defined as *P*_open_ = *k*_op_/(*k*_op_ + *k*_cl_) (36) (Table 2). *P*_open_, by this definition, refers to the kinetic tendency to transition into the open-channel state *versus* reverse itself to the closed-channel state. *P*_open_ was estimated to be 0.94 and 0.86 for GluK2/NETO1 and GluK2/NETO2, respectively, using the respective *k*_op_ and *k*_cl_ values, along with 0.96 for GluK2 receptors without NETO (Table 2). It should be noted that the difference in *P*_open_ between GluK2 with each of the two NETO-containing channels is not statistically significant (Table S7 in Supporting Information). As we previously proposed, potentiation of channel activity is not linked to *P*_open_ (37). Channel potentiation can be induced by nonkinetic parameters alone, such as a higher conductance level and/or a left shift of dose–response relationship (Fig. 2). For NETO-containing channels, a left shift of the dose–response curve was enough to generate a higher receptor response at the same glutamate concentration. Furthermore, all the *P*_open_ values (Table 2) are still considered high or close to unity. Such a high value indicates that the channel-opening rate is still hugely favored once glutamate is bound.

## Discussion

Using a laser-pulse photolysis technique combined with whole-cell recording, we investigated whether NETO1 and NETO2 affected the channel-opening rate process of GluK2 kainate receptors. Our results have revealed that both NETO1 and NETO2 slow the channel-opening and also the channel-closing rates of GluK2. Yet, NETO2 exerts a stronger impact on both *k*_op_ and *k*_cl_ than NETO1 (Table 2). This can be demonstrated by the ratio of the respective rate constants: NETO1 slows the rate of channel opening and closing by no more than twofold. In contrast, NETO2 reduces the channel-closing and channel-opening rate constants by >3-fold and >6-fold, respectively (Table 2). On the millisecond time scale, NETO2 slows the rate of GluK2 channel desensitization by ∼7-fold, as compared with ∼5-fold reduction of the rate by NETO1 (Table 1). In addition, NETO2 causes a much larger left shift of the dose–response curve, which yields ∼6-fold decrease in either *K*_1_ or EC_50_ value *versus* 1.5-fold reduction of either parameter with NETO1. Taken together, NETO2 seems to be a much stronger interacting auxiliary subunit than NETO1, as far as the GluK2 subunit is concerned.

The fact that a NETO protein affects both the rate of channel opening and the channel closing (Table 2) is consistent with the notion that a NETO protein is in contact with the GluK2 subunit and that such a contact is maintained through both the closed-channel and the open-channel states. This is based on the understanding that protein–protein interaction is mediated through contacts between surface residues, and these contacts as well as interfaces define the specificity and strength of interaction (38). Moreover, the major impact of NETO proteins, especially NETO2, is on the rate of the GluK2 channel opening. The magnitude of *k*_op_ reflects the speed of the ligand-induced transition from the closed-channel to the open-channel state (28, 29). The closed-channel state encompasses the unliganded, monoliganded, and diliganded as well as other receptor forms with three or four ligand molecules bound (although our results suggest that binding of two glutamate molecules per receptor is sufficient to induce channel opening). Once an adequate number of ligands are bound, the receptor changes its conformation and undergoes a transition into the open or ion-conducting state. The fact that the *k*_op_ is reduced suggests that the NETO-containing closed-channel state undergoes a slower dynamic change to turn into the open-channel state. Furthermore, both NETO proteins slow the rate of channel closing or reduce *k*_cl_ (Table 2). In other words, NETO-containing GluK2 channels, once opened, stay open longer because they close more slowly than GluK2 channels without NETO. Overall, the fact that NETOs slow both *k*_cl_ and *k*_op_ is consistent with their mode of action through both the closed- and the open-channel states (39, 40), and the consequence of this contact–interaction with GluK2 is to slow the transition from the closed-channel to the open-channel state and prolong the lifetime of the open-channel state.

One obvious possibility that the contact interaction between NETO and GluK2 leads to the reduction of channel-opening kinetics is that NETO proteins, when coassembled with the pore-forming subunit(s), stabilize the structure of the pore-forming subunit GluK2, including perhaps even the unliganded receptor form. In the dynamic context, the stabilization of the receptor would be accompanied by a slower motion toward channel opening and even channel desensitization on the shorter and longer time scales, respectively. This interpretation of our results is consistent with the finding from a cryo-electron microscopic study (41). In the closed-channel state, NETO2 makes extensive contacts with GluK2 in that the N-terminal CUB1 of NETO2 interacts with the lower lobe in the amino terminal domain of the A/C subunit of GluK2, the CUB2 of the NETO2 interacts with the upper lobe of ligand-binding domain (LBD) (*i.e.*, D1-lobe of B/D subunits), and the LDLa domain of the NETO2 interacts with the LBD lower lobe (*i.e.*, D2-lobe of A/C subunits) (41). Certain mutations in GluK2, which disrupt and destabilize some of these contacts, also affect channel desensitization (41). The M3–S2 gating linker and LBD dimer interface are thought to be critical determinants for NETO2 activity (32). Furthermore, the interaction between the N-terminal domain of GluK2 and CUB1 of NETO proteins is critical to NETO-mediated GluK2 activity, and the interaction is electrostatic attraction between positively charged residues in the GluK2 N-terminal domain and negatively charged residues in the CUB1 domain of NETOs (33). It is further interesting to note that transmembrane AMPA receptor regulatory proteins (TARPs) have considerably less contact with their conical AMPA receptors as compared with NETOs to kainate receptors (41, 42, 43, 44). The interaction between TARPs and AMPA receptors involves electrostatic as well as van der Waals interactions (42, 44). Perhaps not surprisingly, as we reported previously (37), TARPs reduce the magnitude of each of these parameters, that is, *k*_op_*, k*_cl_, EC_50,_ and *K*_1_, similar to what NETO proteins do to kainate receptors. The functional impact of TARPs on AMPA receptors is much smaller than the impact of NETO proteins on kainate receptors, however. Together, these results support the notion that the extent to which auxiliary proteins, such as NETOs, interact with the pore-forming subunits plays a significant role in stabilizing the structure of the receptor, thereby affecting the kinetic parameters associated with the channel-opening rate process.

One of the major challenges to measuring the channel-opening kinetics for kainate receptors is the fast channel desensitization that occurs in the millisecond time region, and the reaction is heterogeneous. Our study was made possible because of the use of the laser-pulse photolysis technique. This technique has allowed us to measure the channel-opening rate from an ensemble of channels that are virtually activated in unity. Furthermore, the data collection is completed, for example, within a few milliseconds for GluK2 channels. Therefore, the determination of *k*_op_ and *k*_cl_ is without any significant contribution or “contamination” from channel desensitization. If we take the rise of whole-cell current generated from the laser-pulse photolysis technique where ∼300 μM glutamate was released (Fig. 4), the rate of channel desensitization is ∼130 s^−1^ (Fig. 2); as such, <6% of the rise could be maximally attributed to channel desensitization during the 600 μs rising phase, from which we calculated the *k*_obs_ (*i.e.*, 1880 s^−1^). This estimate assumes that the binding of glutamate could initiate channel desensitization immediately, just as it can initiate channel-opening rate process. It is therefore the maximal percentage of channel desensitization that could possibly “contaminate” the current rise. Yet, we have used the prior ∼95% of the current rise to calculate *k*_obs_ for the determination of *k*_op_ and *k*_cl_. Because we can measure the channel-opening reaction separate from channel desensitization, our model (Fig. 5) and our data analysis to obtain both *k*_op_ and *k*_cl_ do not involve channel desensitization.

Our results (Tables 1 and 2) suggest that NETO2 has a stronger impact on GluK2 channel properties than NETO1, and thus, it is possible that NETO2 could be the preferred accessory protein to associate with GluK2. GluK2 is widely expressed in the central nervous system. For example, GluK2 is abundantly present in pyramidal neurons of the hippocampus, cerebellar granule cells, amygdala, pyriform, anterior cingulate cortexes, and others (45, 46, 47). NETO2 is similarly broadly expressed throughout the mammalian brain (20, 21). In contrast, NETO1 is found at high levels predominantly in the CA3 region of the hippocampus (21, 48). A similar expression pattern for both GluK2 and NETO2 subunits is consistent with the notion that the NETO2 subunit could be commonly associated with the GluK2 pore-forming subunit as the one to modulate GluK2 or GluK2-containing kainate receptor channels. Furthermore, the GluK2 expression is reduced in both the ventral hippocampus and medial prefrontal cortex in NETO2 knockout mice (49). In NETO2-null mice, a 40% decrease in GluK2-containing kainate receptors at the postsynaptic density is observed in the cerebellum, where NETO2 is normally expressed abundantly (50). On the other hand, the NETO2 level in the cerebella of GluK2 homozygous mice is reduced by 60% relative to a GluK2 heterozygous littermate of GluK2 knockout (20). These results further support that NETO2 may be the preferred accessory protein to regulate GluK2-containing neuronal kainate receptors. Additional studies with both NETO proteins on other homomeric and heteromeric kainate receptors are certainly needed to better understand the role of the two NETO proteins.

Two of the most basic parameters in understanding synaptic activity, synaptic activity–induced changes in synaptic efficacy, and synaptic dysfunction in various neurological diseases are excitatory postsynaptic current (EPSC) and miniature EPSC (mEPSC) (51). Both EPSCs (6, 52, 53) and mEPSCs (54) involving kainate receptors have been known to contain a faster AMPA receptor component and a slower one mediated by kainate receptors (24, 25, 55). The dissimilar kinetics that involve both neuronal AMPA and kainate receptors could be better understood based on different channel-opening and channel-closing rate constants between these receptor subtypes. For example, we have previously reported the kinetic influence of either γ-2 or γ-4, two TARP proteins, on *k*_op_ and *k*_cl_ for GluA4 channels (37). Then, a comparison of *k*_op_ and *k*_cl_ for GluA4/γ-2 or γ-4 with GluK2/NETO2 shows that AMPA receptors coassembled with TARPs open their channels 10-fold faster and close their channels 10-fold faster as well than GluK2/NETO2 (37). In other words, the difference in *k*_op_ and *k*_cl_ between these two types of channels coassembled with their respective accessory proteins could be the underlying source of dissimilar contribution to both EPSCs and mEPSCs. Obviously, this comparison is based only on GluK2 and GluA4 homomeric channels complexed with a limited number of their respective auxiliary subunits. However, this comparison demonstrates the utility, where knowing *k*_op_ and *k*_cl_ for various combinations of AMPA and kainate receptors associated with their respective auxiliary subunits would allow one to simulate or reconstruct the magnitude and longevity of synaptic activity presented by EPSCs and mEPSCs. Lastly, knowing the rate constants for both channel opening and channel closing, as we reported, provides the basis of characterizing a potential molecular agent for its mechanism of action on the channel (40, 56). These agents can be small-molecule compounds and/or biological molecules, such as RNA aptamers (40, 56, 57, 58). Characterization of these kinetic mechanisms is required in developing potent and selective agents as well as potential drug candidates that can regulate neuronal kainate receptors.

## Experimental procedures

### Cell culture and receptor expression

The DNA plasmids encoding NETO1 and NETO2 were generously provided by Dr Susumu Tomita. The plasmids were propagated through an *Escherichia coli* host (DH5α) and purified using a Qiagen kit. GluK2Q with and without NETO was transiently expressed in HEK-293S cells using a previously described procedure (34). HEK-293S cells were from the American Type Culture Collection and were cultured in Dulbecco’s modified Eagle’s medium supplemented with 10% fetal bovine serum and 1% penicillin–streptomycin in a humidified incubator at 37 °C and 5% CO_2_. Plasmids were added at a 1:2 weight ratio of GluK2 (4 μg/35 mm dish) to NETO for sufficient heteromeric receptor expression. In the transient transfection of GluK2, green fluorescent protein and large T-antigen were also transiently expressed at a 7:1 and 14:1 plasmid weight ratio, respectively. NBQX was added (final concentration, 1 μM) after transfection of NETO to prevent cell toxicity. Cells were used for recording ∼24 to 48 h after transfection.

### Whole-cell recording and laser-pulse photolysis

The experimental procedure for whole-cell recording of HEK-293 cells was previously described (34). Briefly, electrodes for whole-cell recording and laser-pulse photolysis measurements were made from borosilicate glass capillaries and fire-polished. The pipette solution contained 110 mM CsF, 30 mM CsCl, 4 mM NaCl, 0.5 mM CaCl_2_, 5 mM EGTA, and 10 mM Hepes (pH adjusted to 7.4 by CsOH). The extracellular buffer solution contained 150 mM NaCl, 3 mM KCl, 1 mM CaCl_2_, 1 mM MgCl_2_, and 10 mM Hepes (pH adjusted to 7.4 by NaOH). Green fluorescence in transfected cells was visualized using a Carl Zeiss Axiovert S100 microscope equipped with a fluorescent detection system. Glutamate-induced whole-cell current was recorded using an Axopatch 200B amplifier at a cutoff frequency of 2 kHz for solution flow measurement and 10 to 20 kHz for laser-pulse photolysis measurement by a built-in, four-pole Bessel filter. Data was digitized at a 4 kHz-50 kHz sampling frequency, respectively, by a Digidata 1550B (Molecular Devices). Clampex 11 was used for data acquisition. All recordings were performed with transfected HEK-293S cells voltage clamped at −60 mV and 22 °C. Unless otherwise noted, each data point we presented, except the laser-pulse photolysis measurements, was an average of at least three measurements collected from at least three separate cells. Linear regression and nonlinear fitting were performed using OriginPro 2020 (OriginLab). Standard deviations from the mean are reported unless otherwise noted. Additional analysis associated with nonlinear regression is provided in supporting information.

For the laser-pulse photolysis measurements, 4-methoxy-7-nitroindolinyl–caged-l-glutamate (Tocris) was used. An HEK-293 cell was first equilibrated with up to 1 mM caged glutamate for 300 ms; a Continuum Minilite II pulse Q-switched Nd:YAG laser delivered a single, 5-nanosecond pulse at 355 nm through a fiber optic cable. For each laser-pulse photolysis measurement, at least two free glutamate solutions of a known concentration were used before and after applying the caged glutamate and laser pulse. The whole-cell current amplitude from the photolytically released glutamate was compared with the amplitudes of the free glutamate, with reference to the respective dose–response, in order to calculate the concentration of released glutamate. Both caged and free glutamate were delivered with a U-tube device for fast solution exchange (29, 34). The U-tube device had a time resolution of ∼1 ms (34), which we also used to measure the rate of channel desensitization.

### Data analysis for whole-cell currents and channel-opening rate measurement

The analysis of glutamate-induced kainate receptor response was based on a general, minimal mechanism of channel opening (Figure 5) (29, 34). Based on this mechanism, Equation 2 was derived, where *I*_A_ is the corrected amplitude of macroscopic current, *I*_M_ is the current per mole of receptor, *R*_M_ is the number of moles of receptor on the cell surface, *L* is the ligand concentration, and Ф^−1^ is the channel-opening equilibrium constant. Whole-cell current amplitudes were corrected for receptor desensitization during the rise time as described previously (29, 34). A nonlinear regression was performed to determine the intrinsic equilibrium dissociation constant, *K*_1_ in correlation with the number of ligand molecules, n (see the supporting information).

(1) $$I_{A}=I_{M}R_{M}\;\frac{L^{n}}{L^{n}+{\Phi \left(L+K_{1}\right)}^{n}}$$

In laser photolysis measurement of the channel-opening rate of a kainate receptor, a *k*_obs_ was calculated using Equation 2 from ∼95% of the rising phase of the whole-cell current, which followed a single-exponential rate process for all measurable ligand concentrations. In Equation 2, *I*_*t*_ represents the current amplitude at time *t*, and *I*_*A*_ is the maximum current amplitude.

(2) $$I_{t}=I_{A\;}\left(1-e^{-k_{\text{obs}}t}\right)$$

By varying the concentration of photolytically released glutamate, we collected a series of *k*_obs_. The concentration of each pulse of photolytically released free glutamate was determined, with respect to the dose–response relationship, from at least two control whole-cell responses evoked by free glutamate solutions with known concentrations. The *k*_op_ and *k*_cl_ values were determined using Equation 3.

(3) $$k_{\text{obs}}=k_{\text{cl}}+k_{\text{op}}{\left(\frac{L}{L+K_{1}}\right)}^{n}$$

It should be noted that Equation 3 was also derived from the general mechanism (Fig. 5). In deriving Equation 3, we assumed that the rate of ligand binding was faster than the rate of channel opening. This assumption was corroborated by the observation that the whole-cell current rise followed a first-order rate law (Equation 2) over the entire range of glutamate concentrations. This is consistent with our previous studies on kainate receptors (29, 34). Each data point from laser-pulse photolysis measurement (Fig. 4*D*) was from one cell. Both linear and nonlinear regression were used for analysis of *k*_obs_ *versus* glutamate concentration based on Equation 3.

### Statistical analysis

Welch *t* tests were performed in R to determine if the differences between receptors were statistically significant. An unpaired *t* test of the maximal *k*_des_ data was performed in OriginPro 2020 (OriginLab) to determine the statistical significance. The exact statistical test used for each parameter, n value, and *p* value is indicated in the figure legends. Full numerical outputs can be found in Tables S7*A* and S8.

## Data availability

All data are contained within the article. All raw data supporting the results of this study are available from the corresponding author on request.

## Supporting information

This article contains supporting information (Figs. S1–S7 and Tables S1*A*–S8).

## Conflict of interest

The authors declare that they have no conflicts of interest with the contents of this article.
