# Supplementary material for: A comparative study of NETO1 and NETO2 on channel-opening kinetics of GluK2 kainate receptors
Source: J Biol Chem. 2025 Nov 4;301(12):110888. doi: 10.1016/j.jbc.2025.110888 (PMC12719658; doi:10.1016/j.jbc.2025.110888)
Supplement: Supporting information [file mmc1.docx]

**Supporting Information**

**A comparative study of NETO1 and NETO2 on channel opening kinetics of GluK2 kainate receptors**

**Noah Saunders and Li Niu^1^**

*From the Department of Chemistry and Center for Neuroscience Research, University at Albany, SUNY, Albany, New York 12222*

**_____________________________________________________**

The supporting information contains various data organized in three parts, describe below.

**Part 1 – Use of maximum k_des_ to monitor the ratio of the plasmid of GluK2 to NETOs**

Fig. S1 – The ratio of the plasmid of GluK2 to NETOs vs maximum desensitization rate

**Part 2 – Non-linear regression analysis of dose-response relationships for GluK2, GluK2/NETO1 and GluK2/NETO2**

Here is the summary of the non-linear fitting of the dose-response relationships for the three channel types. Using eq 1, we obtained that dose-response *K_1_* values for GluK2, GluK2/NETO1, and GluK2/NETO2 were estimated to be 300 µM (Table S1C), 210 µM (Table S2C) and 73 µM (Table S3C), respectively. These values were in good agreement with the assumption that the number of ligand molecules bound per receptor, n, for either channel type is around 2. In addition, the same dose-response relationship was also analyzed using the Hill equation by nonlinear regression, and the results are listed in Table 1 in the text.

Table S1A-1E – Detailed data for nonlinear fitting of the dose-response relationship of GluK2 associated with Fig. S2.

Table S2A-2E – Detailed data for nonlinear fitting of the dose-response relationship of GluK2/NETO1 associated with Fig. S3.

Table S3A-3E -- Detailed data for nonlinear fitting of the dose-response relationship of GluK2/NETO2 associated with Fig. S4.

Fig. S2-S4 – Nonlinear regression of the dose-response data using eq 1 with various n values.

Fig. S5-S7 – Nonlinear regression analysis of the dose-response data using Hill equation.

**Part 3 -- Non-linear regression analysis of the channel-opening kinetics for GluK2, GluK2/NETO1 and GluK2/NETO2 obtained using laser-pulse photolysis analysis**

Here is the summary of the non-linear fitting of the k_obs_ vs glutamate concentration data obtained from the laser-pulse photolysis experiment for the three channel types.

First, the *K_1_* values for GluK2, GluK2/NETO1, and GluK2/NETO2 were estimated from eq 3 by nonlinear fitting. These values were found to be ~200-400 µM, ~100-200 µM, and ~40-70 µM, respectively. These values are in good agreement with those obtained from dose-response data as shown in Table 1 in the text.

Second, here is the detailed fitting process. Initially, eq 3 was used to fit the data without any constraints. From this, we found that the best n value was around 2. Next, when the *n* value was fixed as an integer, the use of non-linear regression yielded the *k_cl_* of ~400, ~200, and ~100 s^-1^ for GluK2, GluK2/NETO1 and GluK2/NETO2 (Table S4) respectively. From further fixing both the *n* and *k_cl_* values, the best fits of *k_op_* are 4.2 X 10^3^, 3.0 X 10^3^, and 0.86 x 10^3^ for GluK2, GluK2/NETO1, and GluK2/NETO2, respectively (Tables S4C, S5C, and S6C). All of these values are in good agreement with the linear fit, as shown in Fig 4 and Table 2 in the text.

**Part 4 – Statistical analysis of all measured parameters for GluK2, GluK2/NETO1, and GluK2/NETO2**

Here is the summary of the t-tests used to detect significance in the K_1_, EC_50_, k_op_, k_cl_, P_open_ and k_des_ values between GluK2 and either GluK2/NETO1 or GluK2/NETO2. For all Welch t-tests, two-tailed p values indicate statistical significance of differences. Welch-Satterthwaite degrees of freedom were used. 95% confidence intervals are provided.

First, non-linear fitting of the dose-response relationships using eq 1 and the Hill equation was performed to estimate both the K_1_ and EC_50_, respectively. A detailed summary of the process is described in Part 2. From the means and standard deviations provided, a Welch t-test was conducted in R. The EC_50_ values were found to be significant.

Second, after a non-linear fitting process was used to estimate both the k_op_ and k_cl_, they were then calculated using linear regression from the linear fit, as shown in Fig 4 and Table 2 in the text. A detailed summary of the non-linear fitting is described in Part 3. From the means and standard deviations provided, the P_open_ was calculated and the error was propagated. Welch t-tests of the k_op_, k_cl_, and P_open_ were conducted in R. Between GluK2 and either GluK2/NETO1 or GluK2/NETO2, all k_op_ and k_cl_ values were found to be significant.

Third, the maximal k_des_ values at 10 mM glutamate for GluK2, GluK2/NETO1, and GluK2/NETO2 were analyzed using an unpaired t-test in OriginPro 2020.

Table S7A-7B – Detailed outputs for Welch t-tests of K_1_, EC_50_, k_op_, and k_cl_ values between GluK2 and either GluK2/NETO1 or GluK2/NETO2.

Table S8 – Detailed outputs for unpaired t-tests of the maximal k_des_ values between GluK2 and either GluK2/NETO1 or GluK2/NETO2.

**Part 1 – Use of maximum k_des_ to monitor the ratio of the plasmid of GluK2 to NETOs**

**Figure S1**


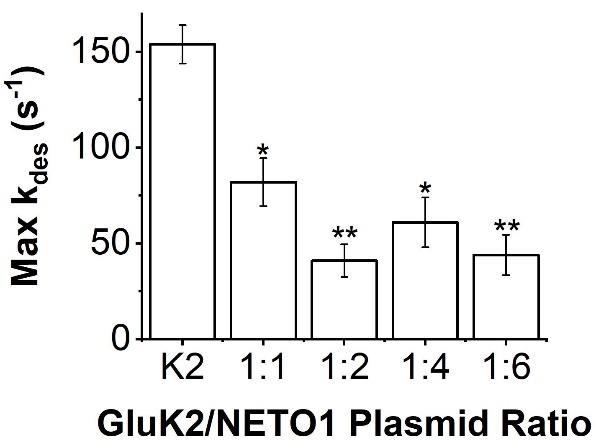


Figure S1. Comparison of the maximum rates of channel desensitization for GluK2 against GluK2/NETO1 at different transfected plasmid ratios. The largest difference in desensitization against GluK2 is observed at the 1:2 ratio and does not further increase with inclusion of more NETO1 plasmid. *p<0.05 from GluK2, **p<0.05 from GluK2 and 1:1 GluK2/Neto1 ratio determined by two tailed Student’s t-test.

**Part 2 – Non-linear regression analysis of dose-response relationships for GluK2, GluK2/NETO1 and GluK2/NETO2**

**Table S1A- Nonlinear fitting of GluK2 dose-response data without constraining any parameter.** Dose-response data was fitted with eq 1 (Methods) with all parameters as outputs. This procedure allowed us to triangulate a trend in all variables. Specifically, *n*, the number of ligand molecules that were sufficient to open the channel appeared to be around 2. The plot was generated using the average values shown at the bottom of this page.

| Initial Values | | | | Outputs | | | | |
| --- | --- | --- | --- | --- | --- | --- | --- | --- |
| I_M_R_M_ | Φ | K_1_ (µM) | n | I_M_R_M_ | Φ | K_1_ (µM) | n | R^2^ |
| 50 | 0.6 | 100 | 1 | 97 | 0.029 | 1580 | 1.7 | 0.999 |
| 50 | 0.5 | 300 | 2 | 151 | 0.47 | 270 | 1.92 | 0.999 |
| 50 | 0.4 | 100 | 1 | 119 | 0.173 | 560 | 1.73 | 0.999 |
| 50 | 0.4 | 300 | 4 | 105 | 0.123 | 330 | 2.55 | 0.997 |
| 50 | 0.5 | 100 | 3 | 128 | 0.41 | 220 | 1.64 | 0.986 |
| 50 | 0.5 | 300 | 4 | 115 | 0.22 | 280 | 2.16 | 0.999 |
| 100 | 0.4 | 100 | 1 | 128 | 0.309 | 270 | 2.08 | 0.999 |
| 100 | 0.4 | 300 | 2 | 121 | 0.274 | 240 | 2.22 | 0.999 |
| 100 | 0.5 | 100 | 4 | 126 | 0.281 | 310 | 1.97 | 0.999 |
| 100 | 0.6 | 300 | 2 | 133 | 0.36 | 240 | 2.12 | 0.999 |
| 100 | 0.4 | 100 | 3 | 131 | 0.334 | 270 | 2.04 | 0.999 |
| 150 | 0.5 | 300 | 1 | 154 | 0.525 | 220 | 2.09 | 0.999 |
| 150 | 0.6 | 100 | 3 | 162 | 0.626 | 180 | 2.24 | 0.999 |
| 150 | 0.6 | 300 | 4 | 148 | 0.488 | 210 | 2.16 | 0.999 |
| 300 | 0.4 | 100 | 2 | 173 | 0.64 | 260 | 1.87 | 0.999 |
| 300 | 0.5 | 300 | 3 | 192 | 0.888 | 150 | 2.28 | 0.999 |
| 300 | 0.6 | 100 | 1 | 202 | 0.926 | 190 | 2.02 | 0.999 |
| 300 | 0.6 | 300 | 2 | 202 | 0.854 | 260 | 1.78 | 0.999 |

|  | I_M_R_M_ | Φ | K_1_ (µM) | n | R^2^ |
| --- | --- | --- | --- | --- | --- |
| Average | 144 | 0.441 | 335.6 | 2.03 |  |
| Final output | 146 ± 88 | 0.42 ± 0.83 | 298 ± 446 | 1.88 ± 0.55 | 0.999 |


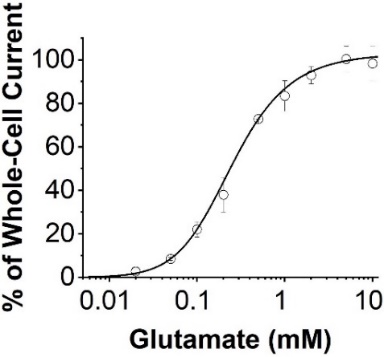


**Table S1B- Nonlinear fitting of GluK2 dose-response data at fixed *n* = 1.** Similar to Table S1A, the fitting was conducted using eq 1. For estimation of *K_1_*, *n*, was fixed at integer values *n*=1-4, based on the assumption that the number of glutamate molecules that can bind to the receptor must be integer.

| Initial Values | | | Outputs | | |
| --- | --- | --- | --- | --- | --- |
| I_M_R_M_ | Φ | K_1_ (µM) | I_M_R_M_ | Φ | K_1_ (µM) |
| 50 | 0.8 | 100 | 292 | 1.22 | 740 |
| 50 | 0.8 | 1000 | 139 | 0.172 | 2150 |
| 50 | 0.2 | 100 | 154 | 0.222 | 2010 |
| 50 | 0.2 | 1000 | 149 | 0.128 | 3590 |
| 50 | 0.4 | 100 | 151 | 0.121 | 3430 |
| 50 | 0.4 | 1000 | 134 | 0.139 | 2490 |
| 150 | 0.8 | 100 | 186 | 0.688 | 630 |
| 150 | 0.8 | 1000 | 209 | 0.58 | 1110 |
| 150 | 0.2 | 100 | 166 | 0.347 | 1340 |
| 150 | 0.2 | 1000 | 154 | 0.251 | 1720 |
| 150 | 0.4 | 100 | 194 | 0.473 | 1270 |
| 150 | 0.4 | 1000 | 170 | 0.383 | 1250 |
| 300 | 0.8 | 100 | 275 | 1.09 | 780 |
| 300 | 0.8 | 1000 | 251 | 0.904 | 860 |
| 300 | 0.2 | 100 | 204 | 0.54 | 1150 |
| 300 | 0.2 | 1000 | 181 | 0.374 | 1490 |
| 300 | 0.4 | 100 | 233 | 0.767 | 940 |
| 300 | 0.4 | 1000 | 207 | 0.56 | 1140 |

|  | I_M_R_M_ | Φ | K_1_ (µM) | R^2^ |
| --- | --- | --- | --- | --- |
| Average | 156.11 | 0.491 | 1560.6 |  |
| Final Output | 196 ± 23 | 0.48 ± 0.73 | 1330 ± 350 | 0.956 |

**
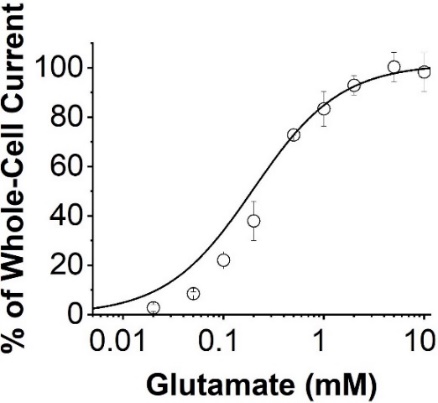
**

**Table S1C- Nonlinear fitting of GluK2 dose-response data at fixed *n* = 2.** This fitting is like that of Table S1B except that *n* was fixed at 2.

| Initial Values | | | Outputs | | |
| --- | --- | --- | --- | --- | --- |
| I_M_R_M_ | Φ | K_1_ (µM) | I_M_R_M_ | Φ | K_1_ (µM) |
| 50 | 0.05 | 100 | 150 | 0.322 | 290 |
| 50 | 0.05 | 1000 | 161 | 0.172 | 370 |
| 50 | 0.2 | 100 | 149 | 0.264 | 320 |
| 50 | 0.2 | 1000 | 108 | 0.146 | 410 |
| 50 | 0.4 | 100 | 124 | 0.265 | 320 |
| 50 | 0.4 | 1000 | 121 | 0.236 | 330 |
| 150 | 0.05 | 100 | 165 | 0.226 | 340 |
| 150 | 0.05 | 1000 | 127 | 0.288 | 310 |
| 150 | 0.2 | 100 | 132 | 0.327 | 290 |
| 150 | 0.2 | 1000 | 161 | 0.167 | 390 |
| 150 | 0.4 | 100 | 149 | 0.467 | 250 |
| 150 | 0.4 | 1000 | 169 | 0.247 | 330 |
| 300 | 0.05 | 100 | 134 | 0.347 | 280 |
| 300 | 0.05 | 1000 | 131 | 0.318 | 290 |
| 300 | 0.2 | 100 | 156 | 0.526 | 240 |
| 300 | 0.2 | 1000 | 118 | 0.216 | 350 |
| 300 | 0.4 | 100 | 183 | 0.762 | 210 |
| 300 | 0.4 | 1000 | 140 | 0.397 | 270 |

|  | I_M_R_M_ | Φ | K_1_ (µM) | R^2^ |
| --- | --- | --- | --- | --- |
| Average | 156.5 | 0.54 | 310.6 |  |
| Final Output | 161 ± 84 | 0.48 ± 0.73 | 300 ± 210 | 0.962 |


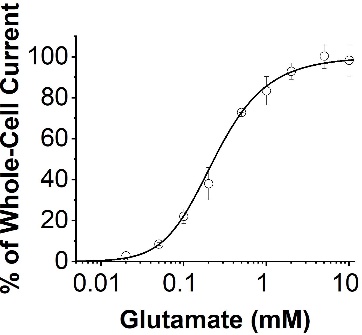


**Table S1D- Nonlinear fitting of GluK2 at fixed *n* = 3.** This fitting is like that of Table S1B except that *n* was fixed at 3.

| Initial Values | | | Outputs | | |
| --- | --- | --- | --- | --- | --- |
| I_M_R_M_ | Φ | K_1_ (µM) | I_M_R_M_ | Φ | K_1_ (µM) |
| 50 | 0.05 | 100 | 156 | 0.315 | 100 |
| 50 | 0.05 | 1000 | 160 | 0.686 | 98 |
| 50 | 0.2 | 100 | 154 | 0.629 | 106 |
| 50 | 0.2 | 1000 | 114 | 0.278 | 135 |
| 50 | 0.4 | 100 | 172 | 0.792 | 97 |
| 50 | 0.4 | 1000 | 130 | 0.415 | 123 |
| 150 | 0.05 | 100 | 117 | 0.299 | 131 |
| 150 | 0.05 | 1000 | 149 | 0.582 | 109 |
| 150 | 0.2 | 100 | 131 | 0.427 | 116 |
| 150 | 0.2 | 1000 | 152 | 0.612 | 115 |
| 150 | 0.4 | 100 | 146 | 0.559 | 95 |
| 150 | 0.4 | 1000 | 133 | 0.44 | 121 |
| 300 | 0.05 | 100 | 122 | 0.345 | 134 |
| 300 | 0.05 | 1000 | 156 | 0.652 | 113 |
| 300 | 0.2 | 100 | 150 | 0.598 | 99 |
| 300 | 0.2 | 1000 | 99 | 0.156 | 162 |
| 300 | 0.4 | 100 | 179 | 0.857 | 101 |
| 300 | 0.4 | 1000 | 149 | 0.59 | 113 |

|  | I_M_R_M_ | Φ | K_1_ (µM) | R^2^ |
| --- | --- | --- | --- | --- |
| Average | 126.2 | 0.314 | 114.9 |  |
| Final Output | 143 ± 43 | 0.52 ± 0.39 | 138 ± 30 | 0.969 |


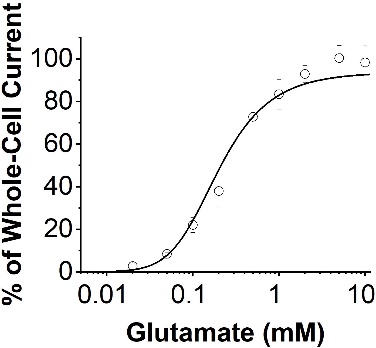


**Table S1E- Nonlinear fitting of GluK2 at fixed *n* = 4.** This fitting is like that of Table S1B except that *n* was fixed at 4.

| Initial Values | | | Outputs | | |
| --- | --- | --- | --- | --- | --- |
| I_M_R_M_ | Φ | K_1_ (µM) | I_M_R_M_ | Φ | K_1_ (µM) |
| 50 | 0.05 | 100 | 149 | 0.638 | 66 |
| 50 | 0.05 | 1000 | 104 | 0.222 | 88 |
| 50 | 0.2 | 100 | 150 | 0.643 | 66 |
| 50 | 0.2 | 1000 | 97 | 0.161 | 97 |
| 50 | 0.4 | 100 | 132 | 0.482 | 71 |
| 50 | 0.4 | 1000 | 117 | 0.252 | 124 |
| 150 | 0.05 | 100 | 115 | 0.323 | 79 |
| 150 | 0.05 | 1000 | 132 | 0.476 | 71 |
| 150 | 0.2 | 100 | 124 | 0.3 | 119 |
| 150 | 0.2 | 1000 | 117 | 0.339 | 78 |
| 150 | 0.4 | 100 | 146 | 0.604 | 67 |
| 150 | 0.4 | 1000 | 118 | 0.349 | 77 |
| 300 | 0.05 | 100 | 149 | 0.635 | 66 |
| 300 | 0.05 | 1000 | 131 | 0.298 | 142 |
| 300 | 0.2 | 100 | 141 | 0.559 | 68 |
| 300 | 0.2 | 1000 | 116 | 0.267 | 113 |
| 300 | 0.4 | 100 | 169 | 0.83 | 62 |
| 300 | 0.4 | 1000 | 120 | 0.369 | 76 |

|  | I_M_R_M_ | Φ | K_1_ (µM) | R^2^ |
| --- | --- | --- | --- | --- |
| Average | 114.89 | 0.229 | 85 |  |
| Final Output | 156 ± 69 | 0.70 ± 0.66 | 60 ± 20 | 0.974 |


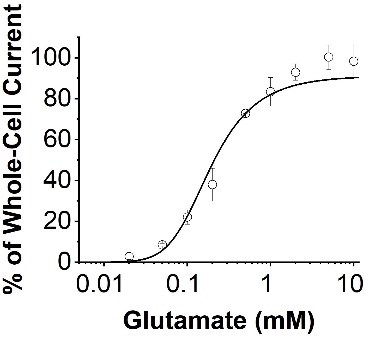


**Table S2A - Nonlinear fitting of GluK2/NETO1 dose-response data with no fixed parameters.** This fitting is like that of Table S1A except that the fitting was done for GluK2/NETO1.

| Initial Values | | | | Outputs | | | | |
| --- | --- | --- | --- | --- | --- | --- | --- | --- |
| I_M_R_M_ | Φ | K_1_ (µM) | n | I_M_R_M_ | Φ | K_1_ (µM) | n | R^2^ |
| 50 | 0.6 | 100 | 1 | 108 | 0.128 | 311 | 2.1 | 0.969 |
| 50 | 0.5 | 300 | 2 | 167 | 0.726 | 104 | 2.4 | 0.96 |
| 50 | 0.4 | 100 | 1 | 133 | 0.379 | 162 | 2.16 | 0.962 |
| 50 | 0.4 | 300 | 4 | 192 | 0.985 | 111 | 2.03 | 0.955 |
| 50 | 0.5 | 100 | 3 | 106 | 0.11 | 164 | 2.48 | 0.93 |
| 50 | 0.5 | 300 | 4 | 109 | 0.13 | 301 | 2.15 | 0.969 |
| 100 | 0.4 | 100 | 1 | 181 | 0.876 | 140 | 1.7 | 0.951 |
| 100 | 0.4 | 300 | 2 | 173 | 0.79 | 121 | 2.03 | 0.956 |
| 100 | 0.5 | 100 | 4 | 137 | 0.423 | 165 | 2.01 | 0.96 |
| 100 | 0.6 | 300 | 2 | 122 | 0.27 | 198 | 2.11 | 0.964 |
| 100 | 0.4 | 100 | 3 | 127 | 0.321 | 195 | 1.98 | 0.961 |
| 150 | 0.5 | 300 | 1 | 192 | 0.98 | 146 | 1.68 | 0.952 |
| 150 | 0.6 | 100 | 3 | 139 | 0.442 | 191 | 1.76 | 0.956 |
| 150 | 0.6 | 300 | 4 | 134 | 0.388 | 199 | 1.85 | 0.958 |
| 300 | 0.4 | 100 | 2 | 114 | 0.192 | 224 | 2.23 | 0.967 |
| 300 | 0.5 | 300 | 3 | 157 | 0.631 | 133 | 2.05 | 0.958 |
| 300 | 0.6 | 100 | 1 | 138 | 0.419 | 137 | 2.41 | 0.963 |
| 300 | 0.6 | 300 | 2 | 192 | 0.982 | 143 | 1.71 | 0.952 |

|  | I_M_R_M_ | Φ | K_1_ (µM) | n | R^2^ |
| --- | --- | --- | --- | --- | --- |
| Average | 146 | 0.51 | 175 | 2.05 |  |
| Final output | 144 ± 5 | 0.49 ± 0.06 | 140 ± 20 | 2.31 ± 0.09 | 0.961 |


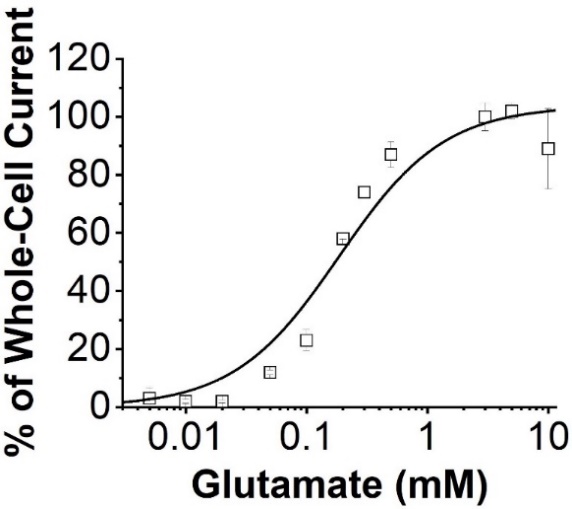


**Table S2B- Nonlinear fitting of GluK2/NETO1 dose-response data at fixed *n* = 1.** This fitting is like that of Table S1B except that the fitting was done for GluK2/NETO1.

| Initial Values | | | Outputs | | |
| --- | --- | --- | --- | --- | --- |
| I_M_R_M_ | Φ | K_1_ (µM) | I_M_R_M_ | Φ | K_1_ (µM) |
| 50 | 0.8 | 100 | 127 | 0.221 | 1031 |
| 50 | 0.8 | 1000 | 126 | 0.211 | 1074 |
| 50 | 0.2 | 100 | 121 | 0.162 | 1330 |
| 50 | 0.2 | 1000 | 114 | 0.102 | 2014 |
| 50 | 0.4 | 100 | 112 | 0.117 | 1403 |
| 50 | 0.4 | 1000 | 117 | 0.161 | 1059 |
| 150 | 0.8 | 100 | 206 | 0.988 | 373 |
| 150 | 0.8 | 1000 | 199 | 0.912 | 390 |
| 150 | 0.2 | 100 | 151 | 0.456 | 590 |
| 150 | 0.2 | 1000 | 135 | 0.299 | 814 |
| 150 | 0.4 | 100 | 172 | 0.658 | 470 |
| 150 | 0.4 | 1000 | 169 | 0.628 | 484 |
| 300 | 0.8 | 100 | 300 | 1.586 | 297 |
| 300 | 0.8 | 1000 | 191 | 0.836 | 414 |
| 300 | 0.2 | 100 | 195 | 0.88 | 406 |
| 300 | 0.2 | 1000 | 120 | 0.158 | 1362 |
| 300 | 0.4 | 100 | 137 | 0.321 | 770 |
| 300 | 0.4 | 1000 | 118 | 0.138 | 1528 |

|  | I_M_R_M_ | Φ | K_1_ (µM) | R^2^ |
| --- | --- | --- | --- | --- |
| Average | 156.11 | 0.491 | 878.3 |  |
| Final Output | 159± 26 | 0.53 ± 0.09 | 544 ± 170 | 0.956 |


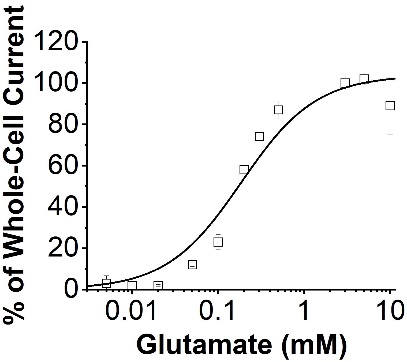


**Table S2C- Nonlinear fitting of GluK2/NETO1 dose-response data at fixed *n* = 2.** This fitting is like that of Table S1C except that the fitting was done for GluK2/NETO1.

| Initial Values | | | Outputs | | |
| --- | --- | --- | --- | --- | --- |
| I_M_R_M_ | Φ | K_1_ (µM) | I_M_R_M_ | Φ | K_1_ (µM) |
| 50 | 0.05 | 100 | 172 | 0.656 | 184 |
| 50 | 0.05 | 1000 | 172 | 0.656 | 184 |
| 50 | 0.2 | 100 | 172 | 0.659 | 183 |
| 50 | 0.2 | 1000 | 172 | 0.656 | 184 |
| 50 | 0.4 | 100 | 177 | 0.7 | 177 |
| 50 | 0.4 | 1000 | 146 | 0.428 | 215 |
| 150 | 0.05 | 100 | 176 | 0.693 | 180 |
| 150 | 0.05 | 1000 | 160 | 0.551 | 194 |
| 150 | 0.2 | 100 | 179 | 0.719 | 177 |
| 150 | 0.2 | 1000 | 151 | 0.473 | 207 |
| 150 | 0.4 | 100 | 170 | 0.686 | 192 |
| 150 | 0.4 | 1000 | 161 | 0.553 | 194 |
| 300 | 0.05 | 100 | 173 | 0.671 | 181 |
| 300 | 0.05 | 1000 | 130 | 0.354 | 186 |
| 300 | 0.2 | 100 | 127 | 0.333 | 154 |
| 300 | 0.2 | 1000 | 126 | 0.308 | 191 |
| 300 | 0.4 | 100 | 141 | 0.459 | 164 |
| 300 | 0.4 | 1000 | 112 | 0.17 | 290 |

|  | I_M_R_M_ | Φ | K_1_ (µM) | R^2^ |
| --- | --- | --- | --- | --- |
| Average | 156.5 | 0.54 | 190.9 |  |
| Final Output | 128 ± 12 | 0.32 ± 0.05 | 210 ± 70 | 0.962 |


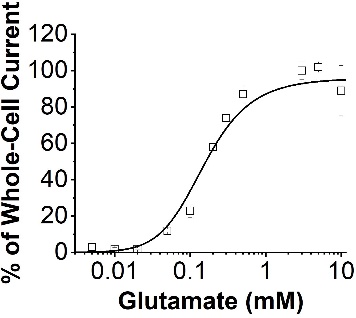


**Table S2D- Nonlinear fitting of GluK2/NETO1 at fixed *n* = 3.** This fitting is like that of Table S1D except that the fitting was done for GluK2/NETO1.

| Initial Values | | | Outputs | | |
| --- | --- | --- | --- | --- | --- |
| I_M_R_M_ | Φ | K_1_ (µM) | I_M_R_M_ | Φ | K_1_ (µM) |
| 50 | 0.05 | 100 | 94 | 0.0069 | 470 |
| 50 | 0.05 | 1000 | 170 | 0.759 | 77 |
| 50 | 0.2 | 100 | 163 | 0.686 | 72 |
| 50 | 0.2 | 1000 | 117 | 0.223 | 132 |
| 50 | 0.4 | 100 | 122 | 0.272 | 118 |
| 50 | 0.4 | 1000 | 106 | 0.108 | 174 |
| 150 | 0.05 | 100 | 126 | 0.309 | 111 |
| 150 | 0.05 | 1000 | 113 | 0.186 | 143 |
| 150 | 0.2 | 100 | 118 | 0.23 | 129 |
| 150 | 0.2 | 1000 | 122 | 0.269 | 117 |
| 150 | 0.4 | 100 | 116 | 0.207 | 131 |
| 150 | 0.4 | 1000 | 126 | 0.315 | 111 |
| 300 | 0.05 | 100 | 134 | 0.392 | 104 |
| 300 | 0.05 | 1000 | 109 | 0.084 | 342 |
| 300 | 0.2 | 100 | 104 | 0.087 | 179 |
| 300 | 0.2 | 1000 | 152 | 0.594 | 75 |
| 300 | 0.4 | 100 | 146 | 0.539 | 77 |
| 300 | 0.4 | 1000 | 134 | 0.393 | 103 |

|  | I_M_R_M_ | Φ | K_1_ (µM) | R^2^ |
| --- | --- | --- | --- | --- |
| Average | 126.2 | 0.314 | 148.1 |  |
| Final Output | 111 ± 6 | 0.17 ± 0.03 | 140 ± 30 | 0.969 |


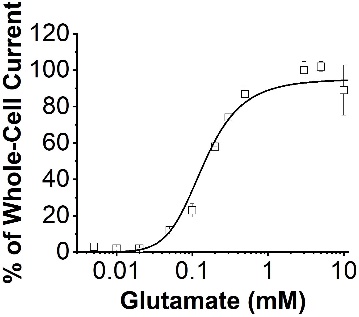


**Table S2E- Nonlinear fitting of GluK2/NETO1 at fixed *n* = 4.** This fitting is like that of Table S1E except that the fitting was done for GluK2/NETO1.

| Initial Values | | | Outputs | | |
| --- | --- | --- | --- | --- | --- |
| I_M_R_M_ | Φ | K_1_ (µM) | I_M_R_M_ | Φ | K_1_ (µM) |
| 50 | 0.05 | 100 | 130 | 0.4 | 58 |
| 50 | 0.05 | 1000 | 128 | 0.34 | 76 |
| 50 | 0.2 | 100 | 115 | 0.217 | 82 |
| 50 | 0.2 | 1000 | 122 | 0.271 | 82 |
| 50 | 0.4 | 100 | 127 | 0.349 | 41 |
| 50 | 0.4 | 1000 | 118 | 0.238 | 86 |
| 150 | 0.05 | 100 | 102 | 0.082 | 140 |
| 150 | 0.05 | 1000 | 112 | 0.171 | 98 |
| 150 | 0.2 | 100 | 113 | 0.184 | 99 |
| 150 | 0.2 | 1000 | 107 | 0.127 | 113 |
| 150 | 0.4 | 100 | 125 | 0.327 | 73 |
| 150 | 0.4 | 1000 | 124 | 0.296 | 80 |
| 300 | 0.05 | 100 | 105 | 0.114 | 122 |
| 300 | 0.05 | 1000 | 97 | 0.0503 | 178 |
| 300 | 0.2 | 100 | 102 | 0.101 | 141 |
| 300 | 0.2 | 1000 | 100 | 0.152 | 149 |
| 300 | 0.4 | 100 | 100 | 0.248 | 99 |
| 300 | 0.4 | 1000 | 141 | 0.472 | 66 |

|  | I_M_R_M_ | Φ | K_1_ (µM) | R^2^ |
| --- | --- | --- | --- | --- |
| Average | 114.89 | 0.229 | 99.1 |  |
| Final Output | 104 ± 5 | 0.10 ± 0.04 | 120 ± 20 | 0.974 |


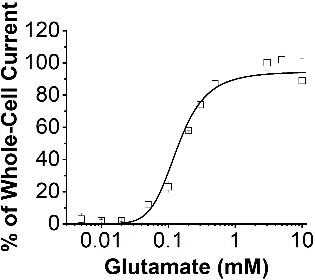


**Table S3A- Nonlinear fitting of GluK2/NETO2 dose-response data without constraining any parameters.** This fitting is like that of Table S1A except that the fitting was done for GluK2/NETO2.

| Initial Values | | | | Outputs | | | | |
| --- | --- | --- | --- | --- | --- | --- | --- | --- |
| I_M_R_M_ | Φ | K_1_ (µM) | n | I_M_R_M_ | Φ | K_1_ (µM) | n | R^2^ |
| 50 | 0.6 | 100 | 1 | 135 | 0.191 | 114 | 2.37 | 0.999 |
| 50 | 0.5 | 300 | 2 | 112 | 0.129 | 102 | 1.96 | 0.999 |
| 50 | 0.4 | 100 | 1 | 114 | 0.204 | 50 | 1.82 | 0.999 |
| 50 | 0.4 | 300 | 4 | 105 | 0.106 | 61 | 2.51 | 0.999 |
| 50 | 0.5 | 100 | 3 | 104 | 0.073 | 116 | 2.19 | 0.999 |
| 50 | 0.5 | 300 | 4 | 122 | 0.197 | 79 | 2.18 | 0.999 |
| 100 | 0.4 | 100 | 1 | 116 | 0.238 | 38 | 1.95 | 0.999 |
| 100 | 0.4 | 300 | 2 | 115 | 0.147 | 96 | 1.96 | 0.999 |
| 100 | 0.5 | 100 | 4 | 115 | 0.155 | 84 | 2.04 | 0.999 |
| 100 | 0.6 | 300 | 2 | 115 | 0.163 | 78 | 2.09 | 0.999 |
| 100 | 0.4 | 100 | 3 | 124 | 0.233 | 64 | 2.17 | 0.999 |
| 150 | 0.5 | 300 | 1 | 148 | 0.388 | 83 | 1.69 | 0.999 |
| 150 | 0.6 | 100 | 3 | 156 | 0.502 | 52 | 1.97 | 0.999 |
| 150 | 0.6 | 300 | 4 | 128 | 0.253 | 76 | 1.92 | 0.999 |
| 300 | 0.4 | 100 | 2 | 153 | 0.496 | 42 | 2.19 | 0.999 |
| 300 | 0.5 | 300 | 3 | 182 | 0.738 | 44 | 1.98 | 0.999 |
| 300 | 0.6 | 100 | 1 | 203 | 0.883 | 51 | 1.79 | 0.999 |
| 300 | 0.6 | 300 | 2 | 173 | 0.708 | 30 | 2.46 | 0.999 |

|  | I_M_R_M_ | Φ | K_1_ (µM) | n | R^2^ |
| --- | --- | --- | --- | --- | --- |
| Average | 134 | 0.322 | 70 | 2.07 |  |
| Final output | 135 ± 2 | 0.31 ± 0.03 | 67 ± 10 | 1.94 ± 0.11 | 0.961 |


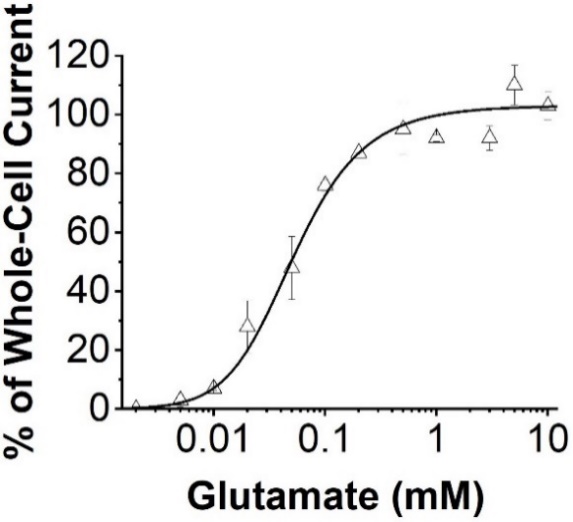


**Table S3B- Nonlinear fitting of GluK2/NETO2 dose-response data at fixed *n* = 1.** This fitting is like that of Table S1B except that the fitting was done for GluK2/NETO2.

| Initial Values | | | Outputs | | |
| --- | --- | --- | --- | --- | --- |
| I_M_R_M_ | Φ | K_1_ (µM) | I_M_R_M_ | Φ | K_1_ (µM) |
| 50 | 0.05 | 100 | 129 | 0.065 | 1292 |
| 50 | 0.05 | 1000 | 124 | 0.022 | 3733 |
| 50 | 0.2 | 100 | 118 | 0.022 | 3061 |
| 50 | 0.2 | 1000 | 102 | 0.047 | 534 |
| 50 | 0.4 | 100 | 104 | 0.016 | 2289 |
| 50 | 0.4 | 1000 | 142 | 0.171 | 541 |
| 150 | 0.05 | 100 | 147 | 0.213 | 448 |
| 150 | 0.05 | 1000 | 129 | 0.065 | 1288 |
| 150 | 0.2 | 100 | 159 | 0.31 | 339 |
| 150 | 0.2 | 1000 | 141 | 0.159 | 583 |
| 150 | 0.4 | 100 | 172 | 0.417 | 271 |
| 150 | 0.4 | 1000 | 153 | 0.26 | 390 |
| 300 | 0.05 | 100 | 170 | 0.402 | 284 |
| 300 | 0.05 | 1000 | 131 | 0.078 | 1106 |
| 300 | 0.2 | 100 | 193 | 0.585 | 215 |
| 300 | 0.2 | 1000 | 147 | 0.213 | 457 |
| 300 | 0.4 | 100 | 217 | 0.787 | 182 |
| 300 | 0.4 | 1000 | 169 | 0.391 | 283 |

|  | I_M_R_M_ | Φ | K_1_ (µM) | R^2^ |
| --- | --- | --- | --- | --- |
| Average | 147.06 | 0.235 | 960.9 |  |
| Final Output | 143 ± 21 | 0.18 ± 0.07 | 523 ± 40 | 0.998 |


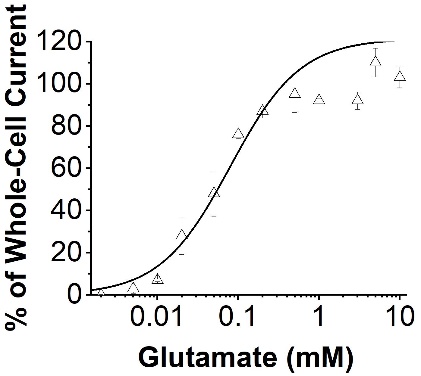


**Table S3C- Nonlinear fitting of GluK2/NETO2 dose-response data at fixed *n* = 2.** This fitting is like that of Table S1C except that the fitting was done for GluK2/NETO2.

| Initial Values | | | Outputs | | |
| --- | --- | --- | --- | --- | --- |
| I_M_R_M_ | Φ | K_1_ (µM) | I_M_R_M_ | Φ | K_1_ (µM) |
| 50 | 0.05 | 100 | 96 | 0.023 | 232 |
| 50 | 0.05 | 1000 | 106 | 0.093 | 109 |
| 50 | 0.2 | 100 | 96 | 0.023 | 228 |
| 50 | 0.2 | 1000 | 96 | 0.022 | 241 |
| 50 | 0.4 | 100 | 140 | 0.379 | 53 |
| 50 | 0.4 | 1000 | 144 | 0.412 | 51 |
| 150 | 0.05 | 100 | 123 | 0.216 | 75 |
| 150 | 0.05 | 1000 | 143 | 0.408 | 51 |
| 150 | 0.2 | 100 | 131 | 0.298 | 60 |
| 150 | 0.2 | 1000 | 147 | 0.446 | 49 |
| 150 | 0.4 | 100 | 146 | 0.431 | 51 |
| 150 | 0.4 | 1000 | 153 | 0.499 | 47 |
| 300 | 0.05 | 100 | 146 | 0.432 | 50 |
| 300 | 0.05 | 1000 | 146 | 0.437 | 52 |
| 300 | 0.2 | 100 | 160 | 0.557 | 45 |
| 300 | 0.2 | 1000 | 102 | 0.052 | 168 |
| 300 | 0.4 | 100 | 168 | 0.63 | 43 |
| 300 | 0.4 | 1000 | 136 | 0.284 | 82 |

|  | I_M_R_M_ | Φ | K_1_ (µM) | R^2^ |
| --- | --- | --- | --- | --- |
| Average | 132.17 | 0.313 | 93.7 |  |
| Final Output | 120 ± 5 | 0.20 ± 0.03 | 73 ± 8 | 0.999 |


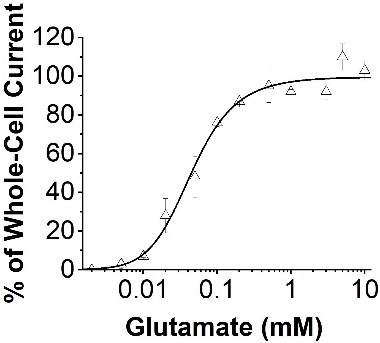


**Table S3D- Nonlinear fitting of GluK2/NETO2 at fixed *n* = 3.** This fitting is like that of Table S1D except that except that the fitting was done for GluK2/NETO2.

| Initial Values | | | Outputs | | |
| --- | --- | --- | --- | --- | --- |
| I_M_R_M_ | Φ | K_1_ (µM) | I_M_R_M_ | Φ | K_1_ (µM) |
| 50 | 0.05 | 100 | 113 | 0.185 | 34 |
| 50 | 0.05 | 1000 | 103 | 0.011 | 323 |
| 50 | 0.2 | 100 | 113 | 0.189 | 33 |
| 50 | 0.2 | 1000 | 155 | 0.574 | 22 |
| 50 | 0.4 | 100 | 113 | 0.185 | 34 |
| 50 | 0.4 | 1000 | 100 | 0.01 | 295 |
| 150 | 0.05 | 100 | 111 | 0.168 | 35 |
| 150 | 0.05 | 1000 | 130 | 0.343 | 27 |
| 150 | 0.2 | 100 | 127 | 0.315 | 27 |
| 150 | 0.2 | 1000 | 112 | 0.18 | 34 |
| 150 | 0.4 | 100 | 142 | 0.454 | 24 |
| 150 | 0.4 | 1000 | 99 | 0.017 | 20 |
| 300 | 0.05 | 100 | 115 | 0.207 | 32 |
| 300 | 0.05 | 1000 | 134 | 0.247 | 60 |
| 300 | 0.2 | 100 | 154 | 0.566 | 22 |
| 300 | 0.2 | 1000 | 127 | 0.258 | 43 |
| 300 | 0.4 | 100 | 182 | 0.829 | 20 |
| 300 | 0.4 | 1000 | 89 | 0.004 | 134 |

|  | I_M_R_M_ | Φ | K_1_ (µM) | R^2^ |
| --- | --- | --- | --- | --- |
| Average | 123.28 | 0.263 | 67.7 |  |
| Final Output | 131 ± 15 | 0.22 ± 0.05 | 55 ± 30 | 0.999 |


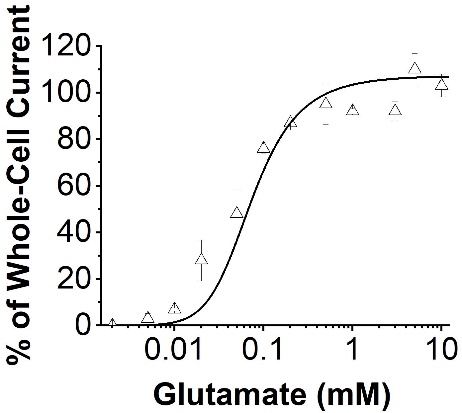


**Table S3E- Nonlinear fitting of GluK2/NETO2 at fixed *n* = 4.** This fitting is like that of Table S1E except that the fitting was done for GluK2/NETO2.

| Initial Values | | | Outputs | | |
| --- | --- | --- | --- | --- | --- |
| I_M_R_M_ | Φ | K_1_ (µM) | I_M_R_M_ | Φ | K_1_ (µM) |
| 50 | 0.05 | 100 | 97 | 0.071 | 28 |
| 50 | 0.05 | 1000 | 100 | 0.093 | 25 |
| 50 | 0.2 | 100 | 113 | 0.09 | 70 |
| 50 | 0.2 | 1000 | 94 | 0.046 | 31 |
| 50 | 0.4 | 100 | 94 | 0.046 | 32 |
| 50 | 0.4 | 1000 | 122 | 0.29 | 18 |
| 150 | 0.05 | 100 | 107 | 0.156 | 22 |
| 150 | 0.05 | 1000 | 115 | 0.222 | 19 |
| 150 | 0.2 | 100 | 128 | 0.338 | 17 |
| 150 | 0.2 | 1000 | 114 | 0.214 | 19 |
| 150 | 0.4 | 100 | 141 | 0.461 | 15 |
| 150 | 0.4 | 1000 | 127 | 0.329 | 17 |
| 300 | 0.05 | 100 | 123 | 0.291 | 18 |
| 300 | 0.05 | 1000 | 116 | 0.228 | 19 |
| 300 | 0.2 | 100 | 151 | 0.559 | 15 |
| 300 | 0.2 | 1000 | 123 | 0.297 | 18 |
| 300 | 0.4 | 100 | 181 | 0.845 | 13 |
| 300 | 0.4 | 1000 | 148 | 0.527 | 15 |

|  | I_M_R_M_ | Φ | K_1_ (µM) | R^2^ |
| --- | --- | --- | --- | --- |
| Average | 121.89 | 0.284 | 22.8 |  |
| Final Output | 123 ± 18 | 0.28 ± 0.16 | 21 ± 5 | 0.999 |


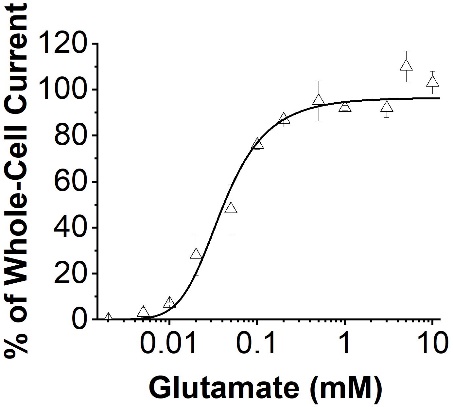


**Figure S2**

**
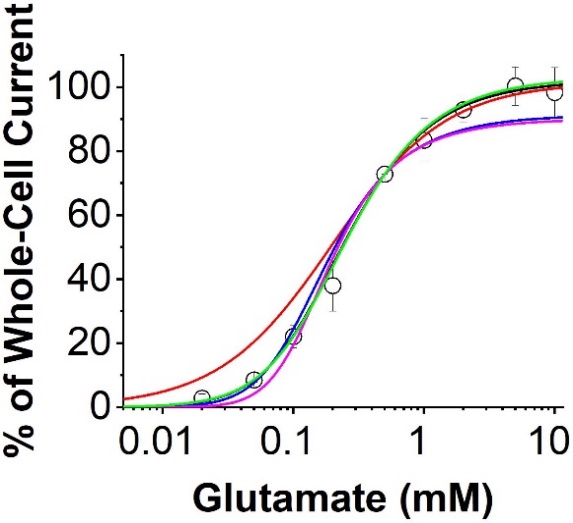
**

Figure S2. Fitting of the dose-response relationship of GluK2 using the final parameters from Tables S1A-E, using eq 1. The percentage of the whole-cell current of GluK2 as a function of glutamate concentration was obtained by using whole-cell recording. Shown here are the fits with all parameters varied and n=1-4, color coded as green (all parameters varied), red (n=1), black (n=2), blue (n=3), and pink (n=4). It should be noted that the black line corresponds to the fit shown in Figure 3.

**Figure S3**

Figure S3. Fitting of the dose-response relationship of GluK2/NETO1 using the final parameters from Tables S2A-E, using eq 1. The percentage of the whole-cell current of GluK2/NETO1 as a function of glutamate concentration was obtained by using whole-cell recording. Shown here are the fits with all parameters varied and n=1-4, color coded as green (all parameters varied), red (n=1), black (n=2), blue (n=3), and pink (n=4). It should be noted that the black line corresponds to the fit shown in Figure 3.

**Figure S4**

Figure S4. Fitting of the dose-response relationship of GluK2/NETO2 using the final parameters from Tables S3A-E, using eq 1. The percentage of the whole-cell current of GluK2/NETO1 as a function of glutamate concentration was obtained by using whole-cell recording. Shown here are the fits with all parameters varied and n=1-4, color coded as green (all parameters varied), red (n=1), black (n=2), blue (n=3), and pink (n=4). It should be noted that the black line corresponds to the fit shown in Figure 3.

**Figure S5**


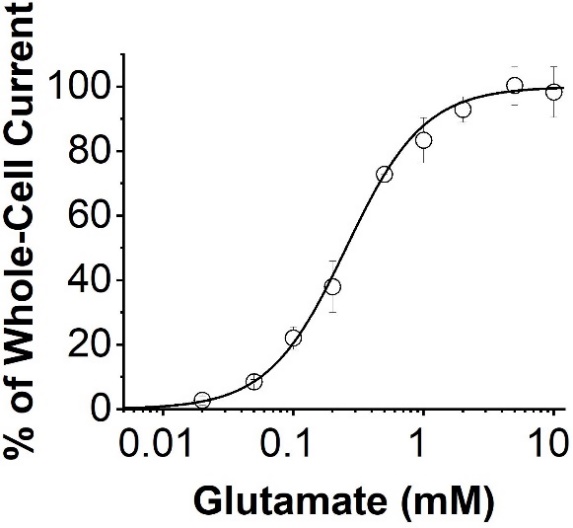


Figure S5. Nonlinear fitting of the dose-response relationship of GluK2 to the Hill equation. The percentage of the whole-cell current of GluK2 as a function of glutamate concentration was obtained by using whole-cell recording. Each data point is an average of at least three measurements from three, separate cells. The solid line is the best fit of the data based on the Hill equation. An *EC_50_* value of 255 ± 30 µM and Hill coefficient of 1.68 ± 0.16 were obtained.

**Figure S6**

**
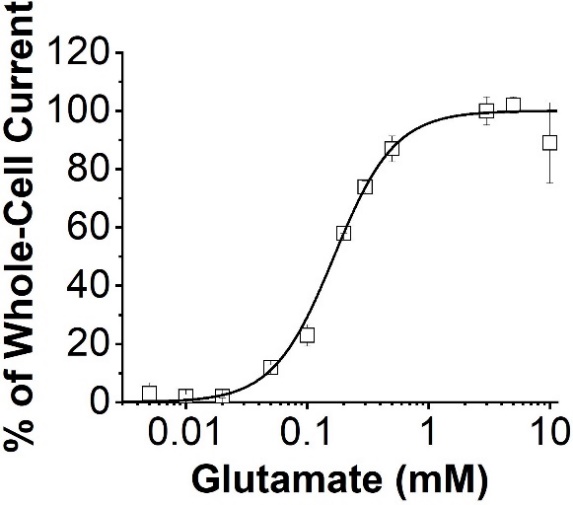
**

Figure S6. Nonlinear fitting of the dose-response relationship of GluK2/NETO1 to the Hill equation. The percentage of the whole-cell current of GluK2/NETO1 as a function of glutamate concentration was obtained by using whole-cell recording. Each data point is an average of at least three measurements from three, separate cells. The solid line is the best fit of the data based on the Hill equation. An *EC_50_* value of 182 ± 17 µM and Hill coefficient of 1.67 ± 0.19 were obtained.

**Figure S7**

**
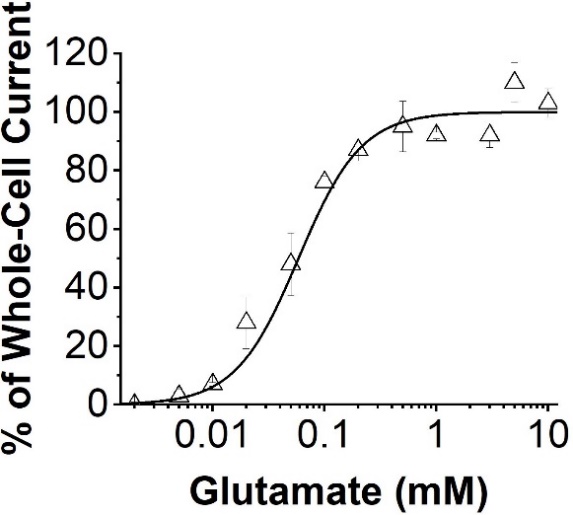
**

Figure S7. Nonlinear fitting of the dose-response relationship of GluK2/NETO2 to the Hill equation. Percentage of the whole-cell current of GluK2/NETO2 as a function of glutamate concentration was obtained by using whole-cell recording. Each data point is an average of at least three measurements from three, separate cells. The solid line is the best fit of the data based on the Hill equation. An *EC_50_* value of 58 ± 4 and Hill coefficient of 1.54 ± 0.08 were obtained.

**Part 3 – Non-linear regression analysis of the channel-opening kinetics for GluK2, GluK2/NETO1 and GluK2/NETO2 obtained using laser-pulse photolysis analysis**

**Table S4A- Nonlinear fitting of *k_obs_* as a function of glutamate concentration for GluK2 to eq 3 without any constraints.** The rate data was fitted with eq 3 (Methods). All parameters were relaxed to allow approximation of *n*, the number of ligand molecules that are bound to the receptor and sufficient to open the channel.

| Initial values | | | | Output | | | | |
| --- | --- | --- | --- | --- | --- | --- | --- | --- |
| k_cl_ (s^-1^) | k_op_ (s^-1^) | K_1_ (µM) | n | k_cl_ (s^-1^) | k_op_ (s^-1^) | K_1_ (µM) | n | R^2^ |
| 50 | 1000 | 50 | 1 | 242 | 5535 | 598 | 1.14 | 0.938 |
| 50 | 1000 | 100 | 2 | 354 | 3905 | 228 | 1.62 | 0.93 |
| 50 | 1000 | 300 | 3 | 328 | 4074 | 256 | 1.61 | 0.935 |
| 50 | 1000 | 200 | 4 | 367 | 3551 | 162 | 2.06 | 0.933 |
| 50 | 7000 | 50 | 1 | 379 | 5699 | 400 | 1.56 | 0.934 |
| 50 | 7000 | 100 | 2 | 332 | 3381 | 169 | 1.8 | 0.918 |
| 50 | 7000 | 300 | 3 | 314 | 4076 | 234 | 1.53 | 0.929 |
| 50 | 7000 | 200 | 4 | 227 | 5600 | 621 | 1.69 | 0.937 |
| 200 | 1000 | 50 | 1 | 360 | 3433 | 175 | 1.84 | 0.925 |
| 200 | 1000 | 100 | 2 | 288 | 4418 | 337 | 1.37 | 0.932 |
| 200 | 1000 | 300 | 3 | 328 | 4070 | 255 | 1.62 | 0.935 |
| 200 | 1000 | 200 | 4 | 370 | 3039 | 114 | 2.34 | 0.928 |
| 200 | 7000 | 50 | 1 | 527 | 7351 | 364 | 2.09 | 0.918 |
| 200 | 7000 | 100 | 2 | 334 | 3382 | 168 | 1.81 | 0.919 |
| 200 | 7000 | 300 | 3 | 277 | 4218 | 317 | 1.39 | 0.936 |
| 200 | 7000 | 200 | 4 | 399 | 3625 | 153 | 2.25 | 0.933 |
| 500 | 1000 | 50 | 1 | 306 | 3771 | 236 | 1.54 | 0.928 |
| 500 | 1000 | 100 | 2 | 320 | 4159 | 278 | 1.5 | 0.926 |
| 500 | 1000 | 300 | 3 | 315 | 4244 | 290 | 1.52 | 0.935 |
| 500 | 1000 | 200 | 4 | 374 | 3530 | 156 | 2.13 | 0.933 |
| 500 | 7000 | 50 | 1 | 406 | 5599 | 557 | 1.34 | 0.92 |
| 500 | 7000 | 100 | 2 | 325 | 3728 | 212 | 1.67 | 0.929 |
| 500 | 7000 | 300 | 3 | 280 | 4539 | 365 | 1.54 | 0.936 |
| 500 | 7000 | 200 | 4 | 394 | 3622 | 155 | 2.21 | 0.933 |

|  | k_cl_ (s^-1^) | k_op_ (s^-1^) | K_1_ (µM) | n | R^2^ |
| --- | --- | --- | --- | --- | --- |
| Average | 339 | 4273 | 283 | 1.72 |  |
| Final output | 280 ± 532 | 4685 ± 7906 | 386 ± 1826 | 1.49 ± 2.52 | 0.935 |


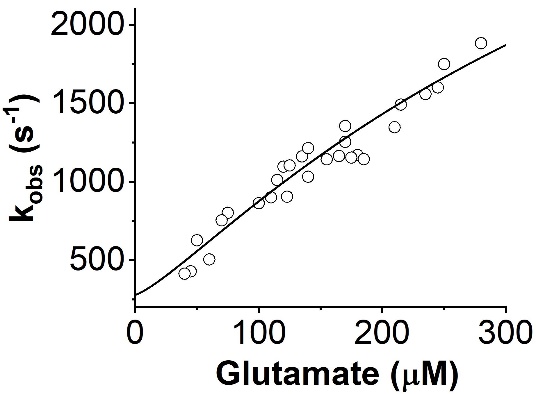


**Table S4B - Nonlinear fitting of *k_obs_* as a function of glutamate concentration for GluK2, when n was fixed at different values.** For the fitting, *n* was fixed at integer values 1 through 4 using eq 3. This assumes each subunit of the tetrameric channel has one glutamate binding site. The fits to *n*=1-4 are color coded as green (*n*=1), red (*n*=2), black (*n*=3), and blue (*n*=4). It should be noted that the red line corresponds to the fit shown in Figure 4.

| Fitted K_1_, k_op_ and k_cl_ with different n values for GluK2 | | | | |
| --- | --- | --- | --- | --- |
| n | k_op_ (x 10^3^ s^-1^) | k_cl_ (s^-1^) | K_1_ (µM) | R^2^ |
| 1 | 9.32 ± 0.94 | 232 ± 97 | 1390 ± 1729 | 0.939 |
| 2 | 3.79 ± 0.86 | 379 ± 82 | 183 ± 61 | 0.933 |
| 3 | 3.22 ± 0.55 | 422 ± 77 | 104 ± 23 | 0.931 |
| 4 | 2.88 ± 0.56 | 508 ± 76 | 72 ± 21 | 0.918 |


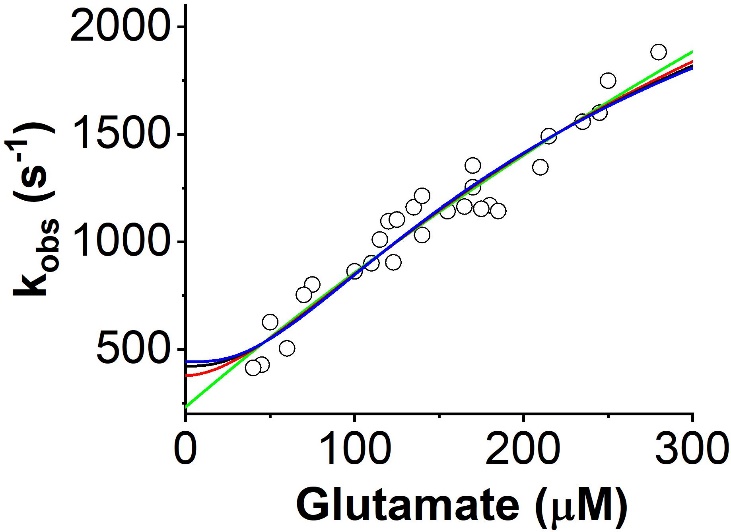


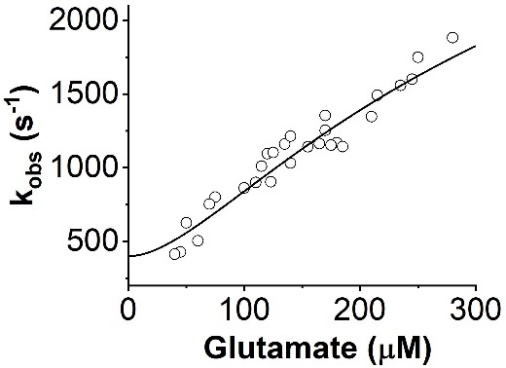
**Table S4C- Nonlinear fitting of *k_obs_* as a function of glutamate concentration for GluK2 to eq 3, when *n* = 2 and *k_cl_* were chosen as different values.** The fitting was similar to Table S3B except that *k_cl_* was fixed at three different values for estimating *k_op_* and *K_1_* using eq 3. The rationale for fixing *k_cl_* was presented in the text. In brief, *k_obs_* ≈ *k_cl_* when *L*<<*K_1_*. The *k_obs_* value at low glutamate concentration for this channel was about 200 s^-1^. The choice of *n* was based on the fitted data from Table S3B, where the *K_1_* value is in closest agreement with the dose-response data at *n* = 2.

| Initial values | | | | Output | | |  |
| --- | --- | --- | --- | --- | --- | --- | --- |
| k_cl_ (s^-1^) | k_op_ (s^-1^) | K_1_ (µM) | n | k_op_ (s^-1^) | K_1_ (µM) | R^2^ |  |
|  |  |  |  |  |  |  |  |
| 50 | 1000 | 100 | 2 | 2624 | 78 | 0.913 |  |
| 50 | 1000 | 300 | 2 | 2608 | 77 | 0.913 |  |
| 50 | 1000 | 600 | 2 | 2623 | 78 | 0.913 |  |
| 50 | 4000 | 100 | 2 | 2640 | 78 | 0.913 |  |
| 50 | 4000 | 300 | 2 | 2560 | 75 | 0.913 |  |
| 50 | 4000 | 600 | 2 | 2566 | 76 | 0.912 |  |
| 50 | 7000 | 100 | 2 | 2728 | 92 | 0.858 |  |
| 50 | 7000 | 300 | 2 | 2645 | 80 | 0.912 |  |
| 50 | 7000 | 600 | 2 | 2382 | 65 | 0.907 |  |
| 400 | 1000 | 100 | 2 | 3979 | 197 | 0.933 |  |
| 400 | 1000 | 300 | 2 | 3974 | 197 | 0.933 |  |
| 400 | 1000 | 600 | 2 | 3956 | 196 | 0.933 |  |
| 400 | 4000 | 100 | 2 | 3923 | 195 | 0.933 |  |
| 400 | 4000 | 300 | 2 | 4431 | 181 | 0.864 |  |
| 400 | 4000 | 600 | 2 | 5498 | 269 | 0.924 |  |
| 400 | 7000 | 100 | 2 | 3860 | 191 | 0.933 |  |
| 400 | 7000 | 300 | 2 | 3982 | 197 | 0.933 |  |
| 400 | 7000 | 600 | 2 | 6867 | 327 | 0.911 |  |
| 600 | 1000 | 100 | 2 | 9717 | 503 | 0.911 |  |
| 600 | 1000 | 300 | 2 | 10565 | 522 | 0.909 |  |
| 600 | 1000 | 600 | 2 | 6918 | 415 | 0.895 |  |
| 600 | 4000 | 100 | 2 | 9027 | 481 | 0.911 |  |
| 600 | 4000 | 300 | 2 | 9522 | 504 | 0.91 |  |
| 600 | 4000 | 600 | 2 | 6131 | 345 | 0.9 |  |
| 600 | 7000 | 100 | 2 | 6627 | 379 | 0.908 |  |
| 600 | 7000 | 300 | 2 | 9070 | 478 | 0.911 |  |
| 600 | 7000 | 600 | 2 | 9684 | 501 | 0.911 |  |

|  | k_cl_ (s^-1^) | k_op_ (s^-1^) | K_1_ (µM) | k_op_ (s^-1^) | K_1_ (µM) | R^2^ |
| --- | --- | --- | --- | --- | --- | --- |
| Final output | 400 | 5226 | 251 | 4162 ± 687 | 200 ± 34 | 0.932 |

^a^Three fixed values of k_cl_ were chosen: 50 s^-1^, 400 s^-1^, and 600 s^-1^

**Table S5A- Nonlinear fitting of k_obs_ as a function of glutamate concentration for GluK2/NETO1 to eq 3, without any constraints.** This fitting is like that of Table S3A, except that it is for GluK2/NETO1.

| Initial values | | | | Output | | | | |  |
| --- | --- | --- | --- | --- | --- | --- | --- | --- | --- |
| k_cl_ (s^-1^) | k_op_ (s^-1^) | K_1_ (µM) | n | k_cl_ (s^-1^) | k_op_ (s^-1^) | K_1_ (µM) | n | R^2^ |  |
|  |  |  |  |  |  |  |  |  |  |
| 50 | 1000 | 50 | 1 | 98 | 2460 | 95 | 2.01 | 0.907 |  |
| 50 | 1000 | 100 | 2 | 93 | 2489 | 105 | 2.08 | 0.961 |  |
| 50 | 1000 | 300 | 3 | 62 | 2592 | 133 | 1.77 | 0.961 |  |
| 50 | 1000 | 200 | 4 | -46 | 2976 | 249 | 1.16 | 0.961 |  |
| 50 | 3000 | 50 | 1 | 62 | 2589 | 131 | 1.77 | 0.96 |  |
| 50 | 3000 | 100 | 2 | 82 | 2524 | 114 | 1.96 | 0.961 |  |
| 50 | 3000 | 300 | 3 | 124 | 2387 | 81 | 2.57 | 0.961 |  |
| 50 | 3000 | 200 | 4 | 72 | 2550 | 120 | 1.86 | 0.961 |  |
| 200 | 1000 | 300 | 1 | 91 | 2520 | 131 | 1.77 | 0.959 |  |
| 200 | 1000 | 200 | 2 | 85 | 2514 | 111 | 1.99 | 0.961 |  |
| 200 | 1000 | 100 | 3 | 120 | 2402 | 84 | 2.49 | 0.961 |  |
| 200 | 1000 | 50 | 4 | 124 | 2389 | 81 | 2.56 | 0.961 |  |
| 200 | 3000 | 300 | 1 | 56 | 2608 | 138 | 1.71 | 0.961 |  |
| 200 | 3000 | 200 | 2 | 25 | 2717 | 167 | 1.5 | 0.961 |  |
| 200 | 3000 | 100 | 3 | 153 | 2300 | 90 | 2.43 | 0.954 |  |
| 200 | 3000 | 50 | 4 | 107 | 2442 | 94 | 2.28 | 0.961 |  |
| 500 | 1000 | 50 | 1 | 139 | 2674 | 134 | 1.9 | 0.954 |  |
| 500 | 1000 | 100 | 2 | 111 | 2428 | 89 | 2.33 | 0.961 |  |
| 500 | 1000 | 300 | 3 | 137 | 2636 | 126 | 1.99 | 0.959 |  |
| 500 | 1000 | 200 | 4 | 106 | 2446 | 95 | 2.26 | 0.961 |  |
| 500 | 3000 | 300 | 1 | 128 | 2423 | 114 | 2.07 | 0.954 |  |
| 500 | 3000 | 200 | 2 | 22 | 2726 | 169 | 1.48 | 0.961 |  |
| 500 | 3000 | 100 | 3 | 100 | 2465 | 99 | 2.18 | 0.961 |  |
| 500 | 3000 | 50 | 4 | 128 | 2374 | 78 | 2.65 | 0.961 |  |

|  | k_cl_ (s^-1^) | k_op_ (s^-1^) | K_1_ (µM) | n | R^2^ |
| --- | --- | --- | --- | --- | --- |
| Average | 91 | 2526 | 117.8 | 2.03 |  |
| Final output | 84 ± 468 | 2525 ± 1494 | 115 ± 387 | 1.96 ± 4.95 | 0.961 |


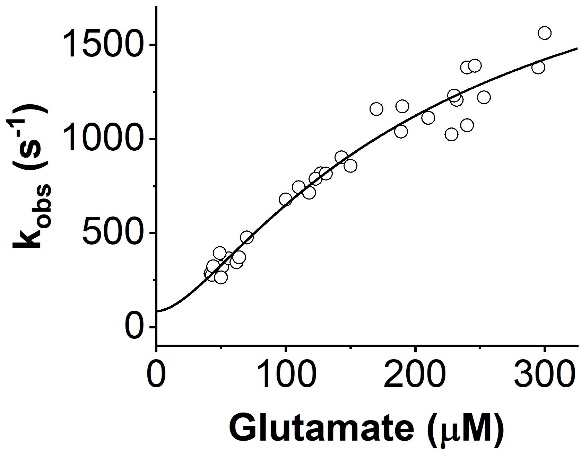


**Table S5B- Nonlinear fitting of k_obs_ as a function of glutamate concentration for GluK2/NETO1, when n was fixed to 1-4.** The fitting routine was similar to the one as in Table S3B. The only exception is that the fitting we did below was for GluK2/NETO1. The fits to n = 1-4 are color coded as green (n=1), red (n=2), black (n=3), and blue (n=4). It should be noted that the red line corresponds to the fit shown in Figure 4.

| Fitted *K_1_*, *k_o_*_p_ and *k_cl_* with different n values for GluK2/NETO1 | | | | |
| --- | --- | --- | --- | --- |
| n | *k_op_* (x 10^3^ s^-1^) | k_cl_ (s^-1^) | *K*_1_ (µM) | R^2^ |
| 1 | 3.18 ± 0.58 | -99 ± 97 | 321 ± 145 | 0.961 |
| 2 | 2.51 ± 0.27 | 86 ± 72 | 165 ± 32 | 0.961 |
| 3 | 2.33 ± 0.21 | 143 ± 65 | 72 ± 17 | 0.96 |
| 4 | 2.24 ± 0.19 | 171 ± 61 | 49 ± 11 | 0.96 |


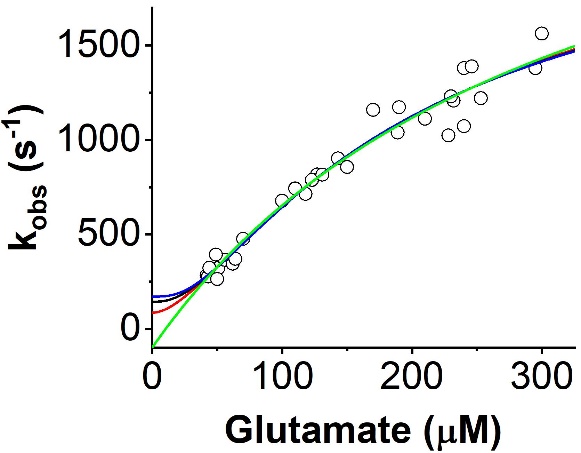


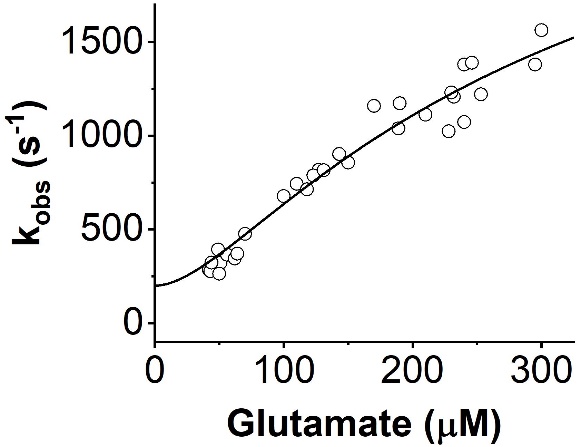
**Table S5C- Nonlinear fitting of k_obs_ as a function of glutamate concentration for GluK2/NETO1, when n = 2 and k_cl_ were fixed at different values.** The fitting was similar to that of Table S3C, except that the fitting was done for the GluK2/NETO1 data.

| Initial values | | | | Output | | |  |
| --- | --- | --- | --- | --- | --- | --- | --- |
| k_cl_ (s^-1^) | k_op_ (s^-1^) | K_1_ (µM) | n | k_op_ (s^-1^) | K_1_ (µM) | R^2^ |  |
|  |  |  |  |  |  |  |  |
| 50 | 3000 | 100 | 2 | 2433 | 104 | 0.961 |  |
| 50 | 3000 | 300 | 2 | 4116 | 197 | 0.91 |  |
| 50 | 3000 | 600 | 2 | 2763 | 139 | 0.9 |  |
| 50 | 4000 | 100 | 2 | 2433 | 98 | 0.961 |  |
| 50 | 4000 | 300 | 2 | 2775 | 142 | 0.92 |  |
| 50 | 4000 | 600 | 2 | 2936 | 157 | 0.85 |  |
| 50 | 5000 | 100 | 2 | 2433 | 105 | 0.961 |  |
| 50 | 5000 | 300 | 2 | 2416 | 103 | 0.961 |  |
| 50 | 5000 | 600 | 2 | 2882 | 146 | 0.86 |  |
| 200 | 3000 | 100 | 2 | 2952 | 158 | 0.966 |  |
| 200 | 3000 | 300 | 2 | 3205 | 181 | 0.963 |  |
| 200 | 3000 | 600 | 2 | 3672 | 212 | 0.955 |  |
| 200 | 4000 | 100 | 2 | 2885 | 154 | 0.96 |  |
| 200 | 4000 | 300 | 2 | 2952 | 159 | 0.957 |  |
| 200 | 4000 | 600 | 2 | 3795 | 223 | 0.941 |  |
| 200 | 5000 | 100 | 2 | 2861 | 15 | 0.955 |  |
| 200 | 5000 | 300 | 2 | 3580 | 204 | 0.953 |  |
| 200 | 5000 | 600 | 2 | 4258 | 246 | 0.93 |  |
| 500 | 3000 | 100 | 2 | 10538 | 699 | 0.849 |  |
| 500 | 3000 | 300 | 2 | 13605 | 794 | 0.858 |  |
| 500 | 3000 | 600 | 2 | 13600 | 791 | 0.858 |  |
| 500 | 4000 | 100 | 2 | 13716 | 822 | 0.855 |  |
| 500 | 4000 | 300 | 2 | 13601 | 784 | 0.857 |  |
| 500 | 4000 | 600 | 2 | 13273 | 776 | 0.857 |  |

|  | k_cl_ (s^-1^) | k_op_ (s^-1^) | K_1_ (µM) | k_op_ (s^-1^) | K_1_ (µM) | R^2^ |
| --- | --- | --- | --- | --- | --- | --- |
| Final output | 200 | 6392 | 309 | 2952 ± 340 | 161 ± 21 | 0.957 |

^a^Three fixed values of k_cl_ were chosen: 50 s^-1^, 200 s^-1^, and 500 s^-1^

**Table S6A- Nonlinear fitting of k_obs_ as a function of glutamate concentration for GluK2/NETO2 to eq 3, without any constraints.** This fitting was similar to that of Table S3A, except that it is for GluK2/NETO2.

| Initial values | | | | Output | | | | |  |
| --- | --- | --- | --- | --- | --- | --- | --- | --- | --- |
| k_cl_ (s^-1^) | k_op_ (s^-1^) | K_1_ (µM) | n | k_cl_ (s^-1^) | k_op_ (s^-1^) | K_1_ (µM) | n | R^2^ |  |
|  |  |  |  |  |  |  |  |  |  |
| 50 | 1000 | 50 | 1 | 45 | 907 | 87 | 0.94 | 0.898 |  |
| 50 | 1000 | 100 | 2 | 131 | 841 | 87 | 1.34 | 0.951 |  |
| 50 | 1000 | 300 | 3 | 126 | 852 | 55 | 1.93 | 0.958 |  |
| 50 | 1000 | 200 | 4 | 243 | 1304 | 124 | 2.49 | 0.904 |  |
| 50 | 3000 | 50 | 1 | -225 | 1162 | 111 | 0.58 | 0.944 |  |
| 50 | 3000 | 100 | 2 | 131 | 841 | 87 | 1.73 | 0.951 |  |
| 50 | 3000 | 300 | 3 | 134 | 810 | 38 | 2.36 | 0.952 |  |
| 50 | 3000 | 200 | 4 | 147 | 833 | 55 | 1.91 | 0.951 |  |
| 100 | 1000 | 300 | 1 | 149 | 823 | 42 | 1.92 | 0.834 |  |
| 100 | 1000 | 200 | 2 | 98 | 840 | 47 | 1.79 | 0.949 |  |
| 100 | 1000 | 100 | 3 | 158 | 785 | 41 | 2.18 | 0.929 |  |
| 100 | 1000 | 50 | 4 | 134 | 800 | 39 | 2.39 | 0.975 |  |
| 100 | 3000 | 300 | 1 | 130 | 959 | 65 | 1.84 | 0.957 |  |
| 100 | 3000 | 200 | 2 | 120 | 856 | 55 | 1.89 | 0.958 |  |
| 100 | 3000 | 100 | 3 | 148 | 870 | 68 | 1.53 | 0.893 |  |
| 100 | 3000 | 50 | 4 | 104 | 837 | 41 | 2.03 | 0.949 |  |
| 300 | 1000 | 50 | 1 | 140 | 772 | 33 | 2.55 | 0.95 |  |
| 300 | 1000 | 100 | 2 | 165 | 865 | 88 | 1.59 | 0.953 |  |
| 300 | 1000 | 300 | 3 | 239 | 897 | 97 | 2.05 | 0.935 |  |
| 300 | 1000 | 200 | 4 | 133 | 881 | 73 | 1.54 | 0.946 |  |
| 300 | 3000 | 300 | 1 | 111 | 836 | 40 | 2.29 | 0.958 |  |
| 300 | 3000 | 200 | 2 | 116 | 856 | 54 | 1.89 | 0.958 |  |
| 300 | 3000 | 100 | 3 | 98 | 861 | 51 | 1.87 | 0.959 |  |
| 300 | 3000 | 50 | 4 | 151 | 808 | 52 | 1.93 | 0.948 |  |

|  | k_cl_ (s^-1^) | k_op_ (s^-1^) | K_1_ (µM) | n | R^2^ |
| --- | --- | --- | --- | --- | --- |
| Average | 122 | 879 | 63.8 | 1.86 |  |
| Final output | 106 ± 112 | 872 ± 257 | 62 ± 124 | 1.68 ± 4.71 | 0.959 |


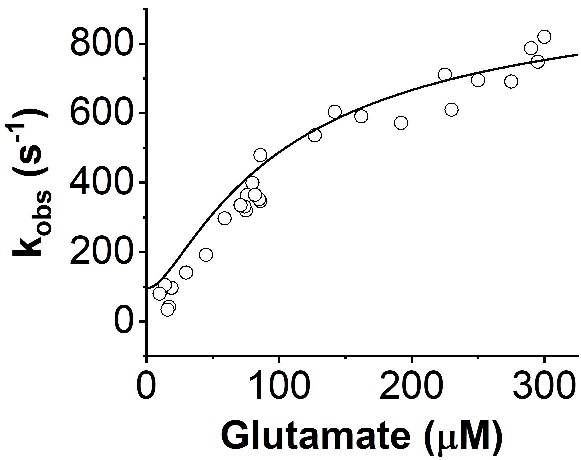


**Table S6B- Nonlinear fitting of k_obs_ as a function of glutamate concentration for GluK2/NETO2, when n was chosen to be 1-4.** The fitting routine was similar to the one as in Table S3B. The only exception is that the fitting we did below was for GluK2/NETO2. The fits to n=1-4 are color coded as green (n=1), red (n=2), black (n=3), and blue (n=4). It should be noted that the red line corresponds to the fit shown in Figure 4.

| Fitted *K_1_*, *k_op_* and *k_cl_* with different n values for GluK2/NETO2 | | | | |
| --- | --- | --- | --- | --- |
| n | k_op_ (x 10^3^ s^-1^) | k_cl_ (s^-1^) | K_1_ (µM) | R^2^ |
| 1 | 0.99 ± 0.05 | 33 ± 58 | 124 ± 42 | 0.96 |
| 2 | 0.85 ± 0.04 | 104 ± 48 | 56 ± 13 | 0.958 |
| 3 | 0.81 ± 0.04 | 125 ± 45 | 34 ± 6 | 0.957 |
| 4 | 0.79 ± 0.04 | 136 ± 43 | 21 ± 4 | 0.957 |


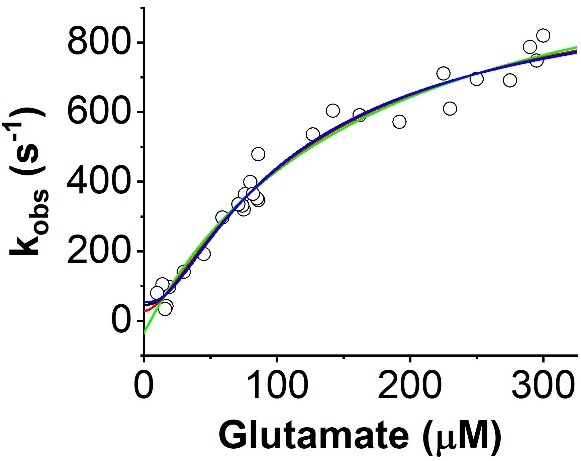


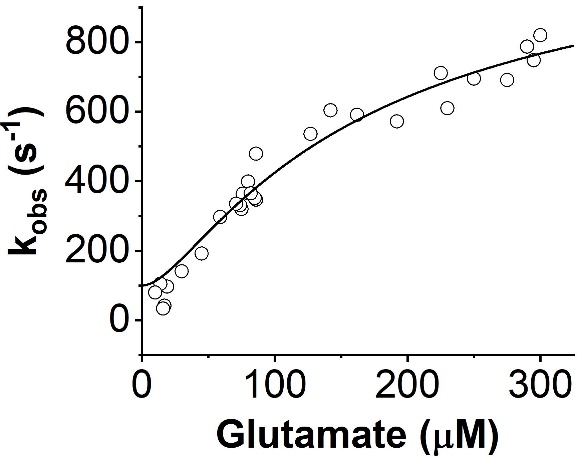
**Table S6C- Nonlinear fitting of k_obs_ as a function of glutamate concentration for GluK2/NETO2, when n =2 and k_cl_ were fixed at different values.** The fitting was similar to that of Table S3C, except that the fitting was done for the GluK2/NETO2 data.

| Initial values | | | | Output | | |  |
| --- | --- | --- | --- | --- | --- | --- | --- |
| k_cl_ (s^-1^) | k_op_ (s^-1^) | K_1_ (µM) | n | k_op_ (s^-1^) | K_1_ (µM) | R^2^ |  |
|  |  |  |  |  |  |  |  |
| 50 | 1000 | 50 | 2 | 870 | 38 | 0.956 |  |
| 50 | 1000 | 100 | 2 | 873 | 38 | 0.956 |  |
| 50 | 1000 | 300 | 2 | 1169 | 65 | 0.854 |  |
| 50 | 2000 | 50 | 2 | 866 | 44 | 0.928 |  |
| 50 | 2000 | 100 | 2 | 866 | 40 | 0.953 |  |
| 50 | 2000 | 300 | 2 | 867 | 39 | 0.955 |  |
| 50 | 3000 | 50 | 2 | 872 | 46 | 0.913 |  |
| 50 | 3000 | 100 | 2 | 815 | 29 | 0.929 |  |
| 50 | 3000 | 300 | 2 | 845 | 36 | 0.955 |  |
| 100 | 1000 | 50 | 2 | 854 | 47 | 0.958 |  |
| 100 | 1000 | 100 | 2 | 977 | 55 | 0.932 |  |
| 100 | 1000 | 300 | 2 | 793 | 41 | 0.952 |  |
| 100 | 2000 | 50 | 2 | 859 | 49 | 0.957 |  |
| 100 | 2000 | 100 | 2 | 852 | 44 | 0.956 |  |
| 100 | 2000 | 300 | 2 | 837 | 45 | 0.958 |  |
| 100 | 3000 | 50 | 2 | 864 | 49 | 0.957 |  |
| 100 | 3000 | 100 | 2 | 822 | 41 | 0.952 |  |
| 100 | 3000 | 300 | 2 | 813 | 43 | 0.956 |  |
| 300 | 1000 | 50 | 2 | 1183 | 176 | 0.903 |  |
| 300 | 1000 | 100 | 2 | 1058 | 145 | 0.896 |  |
| 300 | 1000 | 300 | 2 | 1202 | 174 | 0.902 |  |
| 300 | 2000 | 50 | 2 | 919 | 116 | 0.882 |  |
| 300 | 2000 | 100 | 2 | 1132 | 162 | 0.901 |  |
| 300 | 2000 | 300 | 2 | 1109 | 165 | 0.903 |  |
| 300 | 3000 | 50 | 2 | 890 | 108 | 0.872 |  |
| 300 | 3000 | 100 | 2 | 1113 | 156 | 0.899 |  |
| 300 | 3000 | 300 | 2 | 1162 | 161 | 0.896 |  |

|  | k_cl_ (s^-1^) | k_op_ (s^-1^) | K_1_ (µM) | k_op_ (s^-1^) | K_1_ (µM) | R^2^ |
| --- | --- | --- | --- | --- | --- | --- |
| Final output | 100 | 944 | 80 | 854 ± 76 | 46 ± 9 | 0.958 |

^a^Three fixed values of k_cl_ were chosen: 50 s^-1^, 100 s^-1^, and 300 s^-1^

**Part 4 – Statistical analysis of all measured parameters for GluK2, GluK2/NETO1 and GluK2/NETO2**

**Table S7A- Welch t-tests.** Significance was determined for K_1_, EC_50_, k_op_, k_cl_, and P_open_ values using a Welch t-test in R. The numerical value and standard deviation of each parameter are listed, alongside differences in value and standard deviation of the difference, which were used for calculating the t test.

| Parameter | Comparison | GluK2 | With NETO1 or NETO2 | Difference in Parameter | Standard Deviation of the Difference | t statistic |
| --- | --- | --- | --- | --- | --- | --- |
| K_1_ | GluK2 vs GluK2/NETO1 | 300 ± 210 | 210 ± 70 | 90 | 221.4 | 0.41 |
| K_1_ | GluK2 vs GluK2/NETO2 | 300 ± 210 | 73 ± 8 | 227 | 210.2 | 1.08 |
| EC_50_ | GluK2 vs GluK2/NETO1 | 255 ± 30 | 182 ± 17 | 73 | 34.5 | 2.12 |
| EC_50_ | GluK2 vs GluK2/NETO2 | 255 ± 30 | 58 ± 4 | 197 | 30.3 | 6.51 |
| k_op_ | GluK2 vs GluK2/NETO1 | 5801.91 ± 316.33 | 3536.16 ± 146.50 | 2265.75 | 348.6 | 6.5 |
| k_cl_ | GluK2 vs GluK2/NETO1 | 474.82 ± 39.11 | 217.46 ± 29.93 | 257.36 | 49.22 | 5.23 |
| k_op_ | GluK2 vs GluK2/NETO2 | 5801.91 ± 316.33 | 867.08 ± 40.41 | 4934.84 | 318.9 | 15.48 |
| k_cl_ | GluK2 vs GluK2/NETO2 | 474.82 ± 39.11 | 147.13 ± 18.25 | 327.6 | 43.16 | 7.59 |
| P_open_ | GluK2 vs GluK2/NETO1 | 0.96 ± 0.09 | 0.94 ± 0.15 | 0.02 | 0.18 | 0.11 |
| P_open_ | GluK2 vs GluK2/NETO2 | 0.96 ± 0.09 | 0.86 ± 0.12 | 0.1 | 0.15 | 0.67 |

**Table S7B- Welch t-tests.** Significance was determined for K_1_, EC_50_, k_op_, k_cl_, and P_open_ values using a Welch t-test in R. The numerical value and standard deviation of each parameter are listed, alongside the Welch-Satterthwaite degrees of freedom, two-tailed p value, and 95% confidence interval.

| Parameter | Comparison | GluK2 | With NETO1 or NETO2 | Welch degrees of freedom | p (two-tailed) | 95% CI | Significance |
| --- | --- | --- | --- | --- | --- | --- | --- |
| K_1_ | GluK2 vs GluK2/NETO1 | 300 ± 210 | 210 ± 70 | 29.34 | 0.69 | [-362.5, 542.5] | NS |
| K_1_ | GluK2 vs GluK2/NETO2 | 300 ± 210 | 73 ± 8 | 24.07 | 0.29 | [-206.7, 660.7] | NS |
| EC_50_ | GluK2 vs GluK2/NETO1 | 255 ± 30 | 182 ± 17 | 38.7 | 0.041 | [3.24, 142.76] | Significant |
| EC_50_ | GluK2 vs GluK2/NETO2 | 255 ± 30 | 58 ± 4 | 24.86 | 8.34×10^-7^ | [134.65, 259.35] | Significant |
| k_op_ | GluK2 vs GluK2/NETO1 | 5801.91 ± 316.33 | 3536.16 ± 146.50 | 36.83 | 1.36 × 10⁻⁷ | [1559.3, 2972.2] | Significant |
| k_cl_ | GluK2 vs GluK2/NETO1 | 474.82 ± 39.11 | 217.46 ± 29.93 | 50 | 3.36 × 10^-6^ | [158.49, 356.23] | Significant |
| k_op_ | GluK2 vs GluK2/NETO2 | 5801.91 ± 316.33 | 867.08 ± 40.41 | 26.9 | 6.34 × 10^-15^ | [4280.34, 5589.34] | Significant |
| k_cl_ | GluK2 vs GluK2/NETO2 | 474.82 ± 39.11 | 147.13 ± 18.25 | 36.4 | 5.17 × 10^-8^ | [240.19, 415.18] | Significant |
| P_open_ | GluK2 vs GluK2/NETO1 | 0.96 ± 0.09 | 0.94 ± 0.15 | 46.86 | 0.91 | [-0.33, 0.37] | NS |
| P_open_ | GluK2 vs GluK2/NETO2 | 0.96 ± 0.09 | 0.86 ± 0.12 | 40.83 | 0.51 | [-0.20, 0.40] | NS |

**Table S8- Unpaired t-test .** Significance was determined for maximal k_des_ using unpaired t-tests in OriginPro 2020. The numerical value and standard deviation of each parameter are listed, alongside the n value, degrees of freedom, t statistic, and p value.

| Parameter | Comparison | GluK2 | With NETO1 or NETO2 | n | Degrees of freedom | t statistic | p value | Significance |
| --- | --- | --- | --- | --- | --- | --- | --- | --- |
| k_des_ | GluK2 vs GluK2/NETO1 | 199 ± 12 | 37 ± 6 | 3 | 2 | 27.4 | 1.33×10^-3^ | Significant |
| k_des_ | GluK2 vs GluK2/NETO2 | 199 ± 12 | 27 ± 5 | 3 | 2 | 27.1 | 1.36×10^-3^ | Significant |
